# Posttranslational modification of Aurora A‐NSD2 loop contributes to drug resistance in t(4;14) multiple myeloma

**DOI:** 10.1002/ctm2.744

**Published:** 2022-04-07

**Authors:** Hongmei Jiang, Yixuan Wang, Jingjing Wang, Yafei Wang, Sheng Wang, Enyang He, Jing Guo, Ying Xie, Jingya Wang, Xin Li, Ziyi Peng, Mengqi Wang, Jian Hou, Zhiqiang Liu

**Affiliations:** ^1^ The Province and Ministry Co‐sponsored Collaborative Innovation Center for Medical Epigenetics Tianjin Key Laboratory of Cellular Homeostasis and Human Diseases Department of Physiology and Pathophysiology School of Basic Medical Science Tianjin Medical University Tianjin China; ^2^ Tianjin Medical University Cancer Institute and Hospital National Clinical Research Center for Cancer Tianjin Key Laboratory of Cancer Prevention and Therapy Tianjin's Clinical Research Center for Cancer Tianjin China; ^3^ Department of Hematology Ren Ji Hospital School of Medicine Shanghai Jiao Tong University Shanghai China

**Keywords:** Aurora kinase A, multiple myeloma, NSD2, posttranslational modification

## Abstract

**Background:**

t(4;14)(p16;q32) cytogenetic abnormality renders high level of histone methyltransferase NSD2 in multiple myeloma (MM) patients, and predicts poor clinical prognosis, but mechanisms of NSD2 in promoting chemoresistance have not been well elucidated.

**Methods:**

An epigenetics compound library containing 181 compounds was used to screen inhibitors possessing a prior synergistic effect with bortezomib (BTZ) in vitro. Molecular biology techniques were applied to uncover underlying mechanisms. Transcriptome profile assay was performed by RNA‐seq. NSG mouse‐based xenograft model and intra‐bone model were applied to qualify the synergistic effect in vivo.

**Results:**

We identified an Aurora kinase A inhibitor (MLN8237) possessed a significant synergistic effect with BTZ on t(4;14) positive MM cells. Aurora A protein level positively correlated with NSD2 level, and gain‐ and loss‐of‐functions of Aurora A correspondingly altered NSD2 protein and H3K36me2 levels. Mechanistically, Aurora A phosphorylated NSD2 at S56 residue to protect the protein from cleavage and degradation, thus methylation of Aurora A and phosphorylation of NSD2 bilaterally formed a positive regulating loop. Transcriptome profile assay of MM cells with *AURKA* depletion identified *IL6R, STC2* and *TCEA2* as the downstream target genes responsible for BTZ‐resistance (BR). Clinically, higher expressions of these genes correlated with poorer outcomes of MM patients. Combined administration of MLN8237 and BTZ significantly suppressed tumour growth in LP‐1 cells derived xenografts, and remarkably alleviated bone lesion in femurs of NSG mice.

**Conclusions:**

Aurora A phosphorylates NSD2 at S56 residue to enhance NSD2 methyltransferase activity and form a positive regulating loop in promoting MM chemoresistance, thus pharmacologically targeting Aurora A sensitizes t(4;14) positive MM to the proteasome inhibitors treatment. Our study uncovers a previously unknown reason of MM patients with t(4;14) engendering chemoresistance, and provides a theoretical basis for developing new treatment strategy for MM patients with different genomic backgrounds.

## Graphical abstract

- Aurora A inhibitor MLN8237 sensitizes bortezomib (BTZ) treatment of t(4;14) positive multiple myeloma (MM) cells.
- Methylation of Aurora A and phosphorylation of NSD2 bilaterally form a positive regulating loop to promote MM chemoresistance.
- Heterogeneity of MM patients should be considered when combining Aurora A inhibitors with BTZ in the clinic.

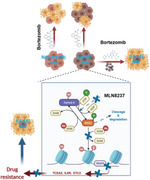

## 1 BACKGROUND

Multiple myeloma (MM) is an aging adults frequently suffering haematological malignancy, characterized by aberrant plasma cells proliferation and ranks the second in all haematological malignancies.^1^ Despite recent innovations for MM treatment, such as proteasome inhibitors (PIs), immunomodulatory agents (IMiDs), monoclonal antibodies (mAbs), cellular therapy chimeric antigen receptor T‐cell immunotherapy (CAR‐T) and autologous stem cell transplantation (ASCT), MM is still incurable due to intrinsic or acquired drug resistance.^2^, ^3^ Hence, it is essential to understand the mechanism of drug resistance in order to overcome treatment failure ultimately.^4^

Chromosomal translocation is one of the characteristics of MM.^5^, ^6^ Of all translocations, the t(4;14)(p16.3;q32.3) is one of the most common cytogenetic abnormalities with an extremely high incidence of 15%,^7^ and closely correlates with poor clinical prognosis.^8^ As a consequence, t(4;14) translocation renders an extra expression of multiple myeloma SET domain containing protein (MMSET, also named *WHSC1/NSD2*) in all patients, and the fibroblast growth factor receptor 3 (*FGFR3*) in about 70% patients, of which only *MMSET* has been proved to be essential to the pathogenesis of MM.^9^, ^10^ High expression of NSD2 is a high risk factor of MM patient for its critical role in altering transcriptome and enhancing DNA damage repair via catalyzing H3K36me2 and H4K20me3, respectively^11^, ^12^, ^13^. NSD2 also methylates none‐histone protein Aurora kinase A and activating NF‐κB signalling pathway to promote the progression of MM.^14^, ^15^ Previous studies have disclosed the clinical significance and roles of NSD2 in MM prognosis, while how NSD2 activity is regulated, and how it is involved in chemoresistance, have not been well elucidated.

High level of Aurora kinase A has been reported in extensive cancers, and its overexpression is associated with poor prognosis in multiple tumours.^16^ Mechanistic studies have indicated that Aurora A induces persistent activation of CDK1 and phosphorylation of SAC and p73 to assist cells in escaping from mitosis and induces genetic instability.^17^ It can also phosphorylate tumour suppressor such as p53 for degradation in tumorigenesis.^18^ Mazzeral et al. reported that Aurora kinase family interacted with the key regulators IκB kinase β (IKKβ) and IKKα to activate NF‐κB pathway in MM,^19^ consequently enhancing drug resistance.^20^ In addition, Aurora A is a target of Wnt/beta‐catenin pathway involved in MM disease progression.^21^ These findings have suggested that Aurora A is a potential target of MM. In animal experiments, an Aurora A selective inhibitor, MLN8237, showed anti‐MM proliferation effect, and promoted apoptosis.^22^ It had potential curative effect in phase I clinical trial study for relapsed or refractory MM patients.^22^, ^23^ However, the application of MLN8237 to MM patients is still controversial in phase II clinical trial study.^24^ Therefore, a better understanding of role and mechanism of Aurora A involved in MM drug resistance is of great value to provide evidence for precise medicine of MM in the future.

In this current study, we screened a compound library targeting epigenetic molecules to identify inhibitors that have synergistic anti‐MM effect with BTZ on MM cells with t(4;14) cytogenetic abnormality, and investigated the role and mechanism of bilateral post translational modifications on Aurora A and NSD2 in MM chemoresistance. The significance and clinical merit of our study is to provide new knowledge of understanding chemoresistance development in MM cells, as well as theoretical basis for personalised treatment in managing MM patients in the clinic.

## 2 METHODS AND MATERIALS

### 2.1 cells and bortezomib‐resistant cells induction

Resources and cultures of HEK‐293T and MM cells, and induction of BR cells have been introduced in our previous report.^25^ The ^WHSC1(+/+)^KMS‐11 and ^WHSC1(+/‐)^KMS‐11 cells were obtained from Horizon Discovery Ltd. (Cambridge, UK). All human cell authentications were identified using short tandem repeat (STR) (Biowing Biotech, Shanghai, China) and mycoplasma‐free was assured using Universal Mycoplasma Detection Kit (ATCC, Manassas, VA, USA).

### 2.2 Epigenetics compound library screening

1 × 10^4^ LP‐1 cells were cultured in each well of 96 well plates, and then treated with BTZ (7 nM) for 48 h. An epigenetics compound library containing 181 inhibitors (Selleck, Catalog No. L1900) (Table S1) was respectively or collaboratively applied on MM cells with gradient concentration of BTZ for 48 h. Then, samples were read at 490 nm by a Microplate Reader 550 (Bio‐Rad Laboratories, Richmond, CA, USA) to calculate cell viability (%) using the formula = treatment group (OD value)/control group (OD value) × 100. Calsyne software was performed to calculate the combination index between BTZ and the inhibitors.

### 2.3 Virus packaging, transfection and infection

Detailed protocols can be found in our previous study.^26^ A ratio of DNA/polyethyleneimine (PEI) (Polysciences, Warrington, PA, USA) = 1:5 in OPTI‐MEM medium (Life Technologies, Carlsbad, CA, USA) was used for transient transfection on HEK‐293T cells in a 10‐cm dish. Plasmids used in this study are listed in the Supporting Information. Harvested supernatant holding corresponding viral particles was concentrated to 100 × volume by poly (ethylene glycol) 8000 (Sigma‐Aldrich, St. Louis, MS, USA). A 50‐μl viral concentrations were added into medium containing 1×10^6^ MM cells with 8 μg/ml polybrene for viral infection.

### 2.4 Flow cytometry analysis

MM cells cultured with or without MLN8237 were administered with BTZ for the indicated time, then Annexin V‐FITC Kit (Sigma‐Aldrich) was applied for apoptosis detection by flow cytometry analysis and FlowJo X software (BD Biosciences, New Jersey, USA) was used to analyze data.

### 2.5 Real‐time polymerase chain reaction, Western blotting and mass spectrum assays

Our previous study has described the protocols of quantitative polymerase chain reaction (qPCR) and western blotting (WB).^25^ Primers for target genes are listed in the supporting information, and the fold change of expression was calculated using the formula of 2^–ΔΔCt^. For WB, cells were lysed with RIPA buffer and then electrophoresed by SDS‐PAGE gel system. After incubation with corresponding primary antibodies as listed in the supporting information and secondary antibodies sequentially, target proteins were visualised by a chemiluminescence system (Millipore, Los Angeles, CA, USA). Coomassie brilliant blue staining was applied before mass spectrum assay.

### 2.6 Co‐immunoprecipitation

Exogenous proteins in HEK293T cells were isolated in NP‐40 buffer and incubated with anti‐FLAG M2 affinity gel (Sigma‐Aldrich). For endogenous protein‐protein interaction in MM cells, the supernatant of cell lysates in NP‐40 buffer was incubated with corresponding antibody following protein G dynabeads at 4°C overnight (ThermoFisher Scientific, Carlsbad, CA, USA). The pellet was dissolved with SDS‐loading buffer and analyzed by WB. Antibodies resources were provided in the Supporting Information.

### 2.7 In vitro direct phosphorylation assay

Briefly, NSD2‐flag protein was purified by co‐immunoprecipitation (Co‐IP) and eluted by 2 × FLAG peptide, and then the protein precipitation dissolution in SDS‐loading buffer was electrophoresed in the phospho‐tag™ SDS‐PAGE (Wako, Tokyo, Japan) under a constant current of 30 mA/gel. Coomassie brilliant blue staining was performed to evaluate the phospho‐NSD2 level by qualifying the shifted and non‐shifted bands. For WB, the gel was washed trice in transfer buffer containing 5 mM EDTA to remove Mn2+ and then the protein was transferred to PVDF membrane, and other protocols were the same as normal WB as described above.

### 2.8 Immunohistochemistry and TUNEL assays

Detailed protocol has been described in our previous report.^26^ Deparaffinized tissue slides were blocked and antigen retrieved, and then appropriately diluted primary antibodies were added onto the slides and incubated in a humidified chamber at 4°C overnight, then appropriately diluted biotinylated secondary antibody was incubated at room temperature for 1 hr. Freshly DAB solution (Dako, K5361) and hematoxylin staining were used to stain indicated antibody and nuclei, respectively. TUNEL assay was carried out according to the DeadEnd™ Fluorometric TUNEL system protocol (Promega, Tokyo, Japan). Briefly, the deparaffinized tissue was rehydrated and incubated with proteinase K. Then, after being fixed with 4% methanol‐free formaldehyde and washed with phosphate buffer saline (PBS), the tissue was incubated with rTdT incubation buffer and terminated with 2 × SSC buffer (Saline Sodium Citrate). At last, the samples were stained with propidium and sealed with Fluoromount‐G for analysis.

### 2.9 The glutathione S‐transferase (GST)‐pulldown assays

For protein purification, GST‐Aurora A was constructed into pGEX‐5X‐3 vector, the plasmid and vector control were transformed into BL21 bacterial cells and the fresh bacterial colony was induced by 0.2 mM IPTG at 16 °C overnight until the colony reached OD_600_ of 0.8‐0.9. Pellets were lysed in GST buffer and centrifuged at 12,000 g at 4 °C for 20 min. Supernatant was added with NaCl (500 mM) and glutathione agarose beads (200 μl) (Thermo Fisher, 16100), and incubated on suspension instrument at 4 °C overnight. The glutathione agarose beads were washed with GST buffer twice next day. Purified protein was evaluated by Coomassie staining.

### 2.10 Chromatin‐immunoprecipitation (ChIP)

Detailed protocol has been described in our previous report.^26^ Briefly, cells were cross‐linked by formaldehyde, quenched by glycine and resuspended in chromatin‐immunoprecipitation (ChIP) lysis buffer, sequentially. After fragmented by ultrasonication with 12 cycles (SONICS, Newtown, CT, USA), chromatin was immunoprecipitated in IP dilution buffer with corresponding antibody and incubated with protein G agarose beads (CST, Danvers, MA, USA). The pellets were washed in TSE buffer (20mMTris, pH8.0, 0.1% SDS, 2 mM EDTA, 1% Triton X‐100), 0.25MLiCl and TE buffer (1mM EDTA, 10mM Tris, pH 8.1) buffer sequentially before elution. At last samples were de‐crosslinked and DNA was purified by QIAquick PCR Purification Kit (QIAGEN, MD, USA).

### 2.11 NOD/SCID mouse xenograft and intra‐bone lesion models

The xenograft (*n* = 6) and intra‐bone models (*n* = 6) were established on NOD.*Cg‐PrkdcscidIl2rgtm1Wjl*/SzJ as before.^27^, ^28^ After indicated time, mice were treated with BTZ (i.p., 0.5 mg/kg) and MLN8237 (5 mg/kg, i.g., with β‐cyclodextrin) solo or together every 2 days. For xenograft models, mice were weighed and tumours were measured every 2 days. Micro computed tomography (CT) analysis with Skyscan 1172 microtomograph (BrukermicroCT, Kontich, Belgium), three‐dimensional (3D) models reconstruction with a surface‐rendering program (Ant, release 2.0.5, Skyscan) and 3D measurements with the CtAn software (release 2.5, Skyscan) were performed on the intra‐bone models. The corresponding parameters including trabecular separation, trabecular volume (BV/TV, in %), cortical thickness and trabecular number (Tb.N, in mm‐1) were calculated on the femur body of intra‐bone model.

### 2.12 Statistical analysis

Three independent and repeated experiments were performed for mean ± SD calculation. Paired two‐tailed Student's *t*‐test and two‐way ANOVA were used for significant analysis of differences between groups. The correlations of gene expressions were analyzed by Pearson correlation test, and GraphPad Prism 5.0. was used to analyze survival analysis along with log‐rank test. The differences were statistically significant only when *p* value was < .05. *, *p *≤ .05; **, *p *≤ .01; ***, *p *≤ .001 compared with the corresponding control, respectively.

## 3 RESULTS

### 3.1 Aurora kinase A inhibitor exerts synergistic effect with BTZ on MM cells

**FIGURE 1:**
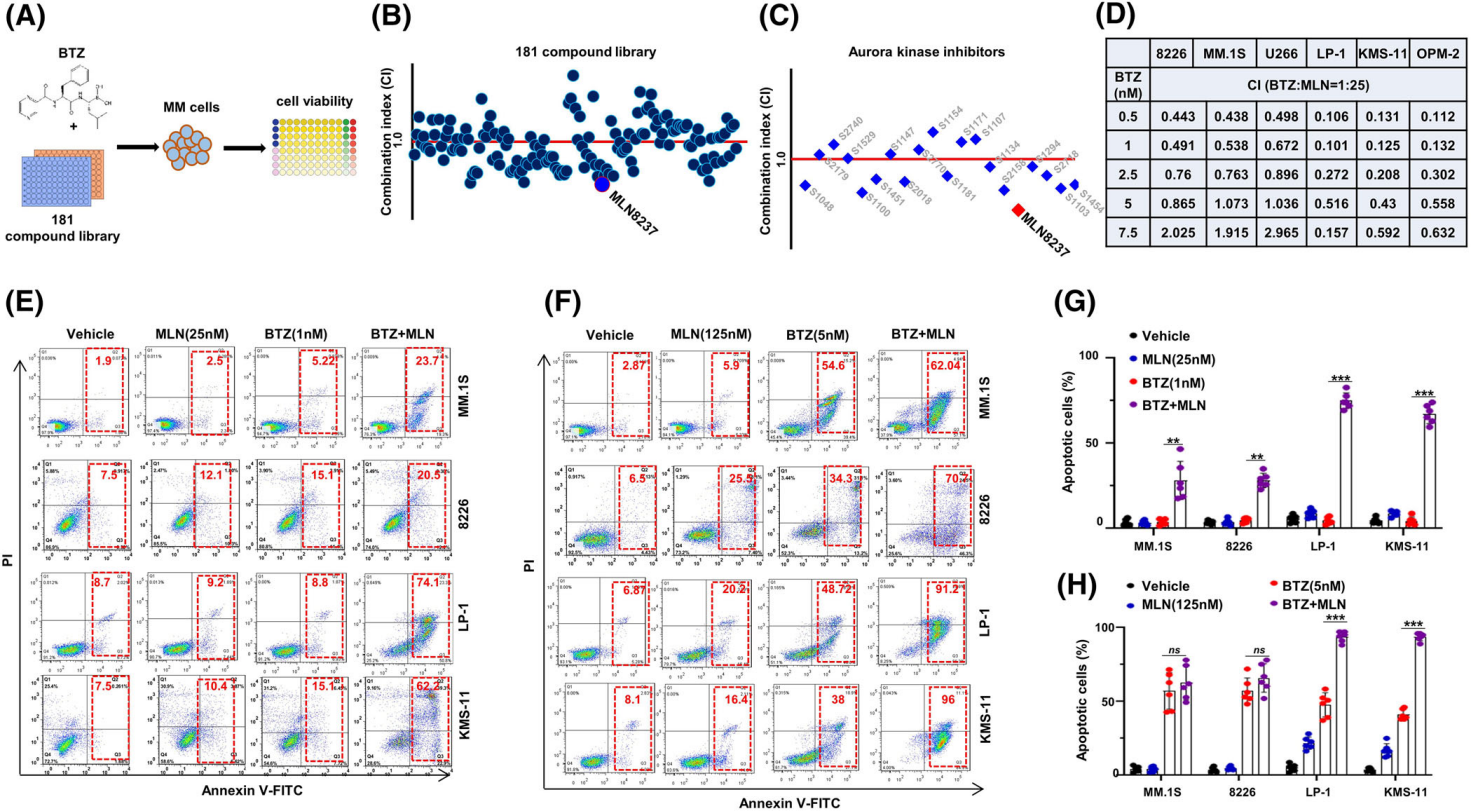
Epigenetics Compound Library screening identifies Aurora A inhibitor as a synergistic effecter with BTZ on t(4;14) MM cells. (A) Schematic diagram illustrates the workflow of Epigenetics Compound Library screening. Inhibitors were administered at three gradient concentrations (5, 50, 500 nM) with 5 nM of bortezomib (BTZ) in LP‐1 cells. (B) Combination index (CI) of BTZ and small molecular inhibitors. CI is a quantitative measure of the degree of drug interaction in terms of additive effect (CI = 1), synergism (CI < 1) or antagonism (CI > 1) for a given endpoint of the effect measurement. (C) The synergetic anti‐multiple myeloma (MM) effects (CI values) of all Aurora kinase family specific inhibitors and BTZ. (D) CI values of 8226, MM.1S, U266, LP‐1, KMS‐11 and OPM‐2 cells treated with different concentration of BTZ and MLN8237 (25×BTZ dosage). (E‐F) Representative flow cytometry assay (*n* = 3) showing apoptotic cells in the 8226, MM.1S, LP‐1 and KMS‐11 cells induced by different concentration of BTZ and MLN8237 (25×BTZ dosage) for 48 h, and (G‐H) shown the statistical analysis of flow cytometry (*n* = 6). Error bars indicate the means ± SD from three biologically independent experiments with each sample triplicated. ** *p* < .01; *** *p* < .001

To discover potential inhibitors that can sensitize BTZ treatment, we screened an epigenetics compound library, containing 181 compounds, on LP‐1 cells, known MM cells with t(4;14) translocation (Figure 1A). When the gradient concentration of each inhibitor was collaboratively applied with BTZ to MM cells, it was discovered that a few inhibitors had synergistic effect (Figure 1B). Besides Histone deacetylase inhibitors, elucidated by our group before,^25^ Aurora kinase A selective inhibitor (MLN8237) was one of the inhibitors that presented the most prominent synergistic effect with BTZ on MM cells (Figure 1C and Figure S1A,B). Interestingly, the synergistic effect of MLN8237 with BTZ was not remarkable on t(4;14) negative MM cells, including 8226, MM.1S and U266, compared with t(4;14) positive MM cells including LP‐1, KMS‐11 and OPM‐2 (Figure 1D and Figure S1C). Through flow cytometry assay, we observed the combination treatment of MLN8237 and BTZ induced more apoptosis of LP‐1 and KMS‐11 cells compared with MM.1S and 8226 cells (Figure 1E,F), and the differences were even more significant in the t(4;14) positive MM cells, either with lower dose (1nM) or higher dose (5nM) of BTZ (Figure 1G,H). Collectively, these findings indicate that Aurora kinase A selective inhibitor is prone to engender synergistic effect on t(4; 14) MM cells.

### 3.2 Aurora A and NSD2 protein levels have positive correlation in t(4;14) positive MM cells

**FIGURE 2:**
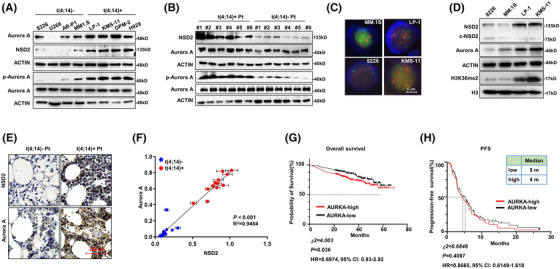
Aurora A and NSD2 levels are positive correlated in t(4;14) MM cells. (A) Representative images of western blotting (*n* = 3) showing NSD2, Aurora A and p‐Aurora A protein levels in t(4;14) positive multiple myeloma (MM) cells and t(4;14) negative MM cells. (B) Representative images of western blotting (*n* = 2) showing NSD2, Aurora A and p‐Aurora A protein levels in CD138^+^ plasma cells derived from t(4;14) positive and t(4;14) negative MM patients. (C) FISH assay showing t(4;14) translocation in 8226, MM.1S, LP‐1 and KMS‐11 cells. Scale bar, 5 μm. Green fluorescent labels IgH(14q32) and red fluorescent labels FGFR3(4p16), orange indicates fusion gene of IgH and *FGFR3*. (D) Representative images of western blotting (*n* = 3) showing NSD2, Aurora A and H3K36me2 levels in 8226, MM.1S, LP‐1 and KMS‐11 cells. (E) Representative immunohistochemical staining (*n* = 12) of Aurora A and NSD2 proteins in the bone marrow slides from t(4;14) positive MM patients and t(4;14) negative MM patients and (F) shown the correlation between NSD2 and Aurora A protein level in t(4;14) positive MM patients and t(4;14) negative MM patients. (G) Kaplan‐Meier survival curve for overall survival of patients with low and high Aurora A expression level in database GSE2658 (cutoff, median). (H) Disease‐free survival (PFS) of patients with low and high Aurora A expression level in database GSE9782 (cutoff, median)

Since t(4;14) translocation engenders high NSD2 expression, we speculated that NSD2 may be involved in engendering synergistic effect between MLN8237 and BTZ on t(4;14) positive MM cells. Thus we detected the expression of Aurora A and NSD2 in four t(4;14) positive and four t(4;14) negative MM cells, and found both Aurora A and NSD2 were obviously higher in the t(4;14) positive MM cells, especially the phosphorylation level of Aurora A (p‐Aurora A) (Figure 2A). Meanwhile, the correlation analysis showed that they were significantly positive correlated (Figure S2A), which was further confirmed by the data extracting from ONCOMINE with the “Single Gene Analysis” module of GEPIA (Figure S2B,C). Of note, CD138^+^ plasma cells derived from MM patients with t(4;14) cytogenetic abnormality showed remarkable Aurora A, p‐Aurora A and NSD2 levels compared with those without t(4;14) (Figure 2B). To clarify whether *AURKA* is a direct target of NSD2 via H3K36me2 modification, we checked the enrichment of H3K36me2 at promoter of AURKA in our previous ChIP‐seq data.^26^ However, we did not find obvious enrichment of H3K36me2 at *AURKA* promoter (Figure S2D). In the isogenic NSD2‐high (NTKO) and NSD2‐low (TKO) KMS‐11 cells,^29^ the mRNA level of AURKA was only slightly downregulated by NSD2 depletion, but the protein level was dramatically suppressed (Figure S2E,F). Based on these results, we speculate that overexpression of Aurora A maybe due to posttranslational regulation of NSD2. In t(4;14) positive LP‐1 and KMS‐11 cells, but not in t(4;14) negative MM.1S and 8226 cells, as shown by in situ hybridization (FISH) assay (Figure 2C), the H3K36me2 level was remarkably elevated along with high levels of NSD2 and Aurora A (Figure 2D). In line with this evidence, patients with t(4;14) cytogenetic abnormity rendered both higher NSD2 and Aurora A level than none‐t(4;14) patients as measured by immunohistochemistry assay (Figure 2E), indicating Aurora A level positively correlated with NSD2 (Figure 2F). To exclude the effect of cell cycle on expressions of NSD2 and Aurora A, we synchronised all MM cells and found no significant difference between Aurora A and NSD2 in starvation condition compared with normal status (Figure S2G), indicating an independent relationship of NSD2 and Aurora A with cell cycle in MM cells. Notably, Aurora A expression predicted a negative association trend with patient's overall survival and disease‐free survival in the cohort GSE2658 and GSE9782, respectively (Figures 2G,H), and the correlations were significant (*p *< .001) in the cohort GSE2658 and GSE4581 (Figure S2H,I). Of note, Aurora A expression was also negatively associated with overall survival and disease‐free survival in patients suffering other haematopoietic malignancies (Figure S2J,K). These findings suggest that Aurora A and NSD2 are positively correlated in MM cells with t(4;14) abnormality, which may be responsible for the difference in sensitivity to combination of BTZ and MLN8237.

### 3.3 Aurora A stabilizes NSD2 and enhances NSD2 methyltransferase activity

**FIGURE 3:**
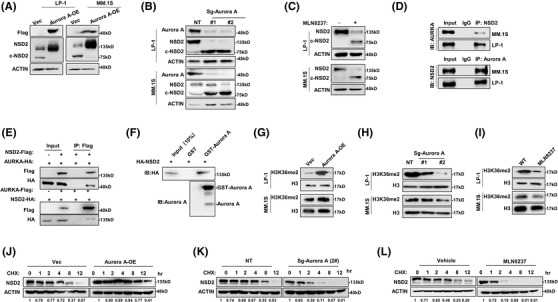
Aurora A interacts and stabilizes NSD2 protein. (A) Representative western blotting (*n* = 3) showing NSD2 level in LP‐1 and MM.1S cells infected with lentivirus carrying pITA‐AURKA‐Flag (Aurora‐OE) compared to the Vector control (Vec) for 48 h. (B) Representative western blotting (*n* = 3) showing NSD2 level in LP‐1 and MM.1S cells infected with lentivirus carrying sgRNA targeting *AURKA* gene (Aurora A‐KO) compared to the non‐target control (NT Ctrl). (C) Representative western blots (*n* = 3) showing the NSD2 level in LP‐1 and MM.1S cells treated with MLN8237 (100 nM) for 6 h. (D) Representative images (*n* = 3) of Co‐IP showing endogenous interaction between Aurora A and NSD2 in LP‐1 and MM.1S cells. (E) Representative images (*n* = 3) of Co‐IP showing exogenous interaction between Aurora A and NSD2 in HEK‐293T cells transfected with pLV‐NSD2‐Flag and pITA‐Aurora A‐HA, and pCMV3‐NSD2‐HA and pITA‐Aurora A‐Flag reversely. (F) GST‐pulldown assay for the GST‐Aurora A fusion protein and HA‐NSD2 with HA antibody (Top) and the Aurora A antibody (bottom) in HEK‐293T cells. Representative western blotting (*n* = 3) showing H3K36me2 level in Aurora A‐OE (G), Aurora A‐KO(H) and (I) MLN8237 (100 nM‐6 h) treated LP‐1 and MM.1S cells. Degradation of NSD2 protein in Aurora A‐OE (J), Aurora A‐KO (K) and (L) MLN8237 (100 nM‐12hr) treated MM cells in the presence of 20 μM cycloheximide (CHX) for up to 12 h

To functionally validate these findings, we exogenously manipulated Aurora A expression either by lentivirus carrying overexpression of AURKA (Aurora A‐OE), or knockout the gene with CRISPR/cas9‐AURKA sgRNA (Aurora A‐KO), and found NSD2 protein level remarkably elevated or suppressed accordingly. Intriguingly, the cleavage of NSD2 almost eliminated due to Aurora A overexpression, but augmented remarkably according to Aurora A depletion, both independent of genomic background (Figure 3A,B). Moreover, pharmacologically blockage of Aurora A activity by the inhibitor MLN8237 at the effective concentration (Figure S3A) successfully inhibited NSD2 protein level (Figure 3C), but not at transcriptional level (Figure S3B). As expected, both endogenous and exogenous immunoprecipitation validated the interaction between Aurora A and NSD2 (Figure 3D,E), and the direct physical interaction between Aurora A and NSD2 was confirmed by in vitro GST‐pulldown assay (Figure 3F and Figure S3C). Since NSD2 exerts its function mainly through methyltransferase activity, we examined whether dimethylation  at histone 3 lysine 36 (H3K36me2) could be affected by Aurora A. Indeed, we found that methyltransferase activity of NSD2 was altered by Aurora A expression accordingly, as H3K36me2 level obviously augmented in Aurora A‐OE MM cells, and remarkably suppressed in Aurora A‐KO MM cells, respectively (Figure 3G,H). Notably, MLN8237 treatment also mimicked the same effect as Aurora A‐KO, and the regulation in NSD2 activity was more obvious in LP‐1 cells than MM.1S cells (Figure 3I). Next, we evaluated whether Aurora A could protect NSD2 protein from degradation. As predicted, NSD2 degradation was significantly retarded due to Aurora A overexpression (Figure 3J and Figure S3D), but remarkably accelerated either by Aurora A depletion or by administration of MLN8237 (Figure 3K,L and Figure S3E,F). As expected, gain‐of‐function of Aurora A suppressed, and pharmacologically abolishment of Aurora A activity augmented the ubiquitination status of NSD2 (Figure S3G). Moreover, we found that kinetic ubiquitination and cleavage of NSD2 were closely correlated (Figure S3I and S3H). We further clarified which type of ubiquitin chain was linked onto NSD2 protein, including K6, K11, K27, K29, K33, K48 and K63, and found NSD2 was mainly modified by K11‐ and K29‐linked ubiquitin chain (Figure S3J,K). All the results suggest that Aurora A could affect the degradation of NSD2 by ubiquitination.

**FIGURE 4:**
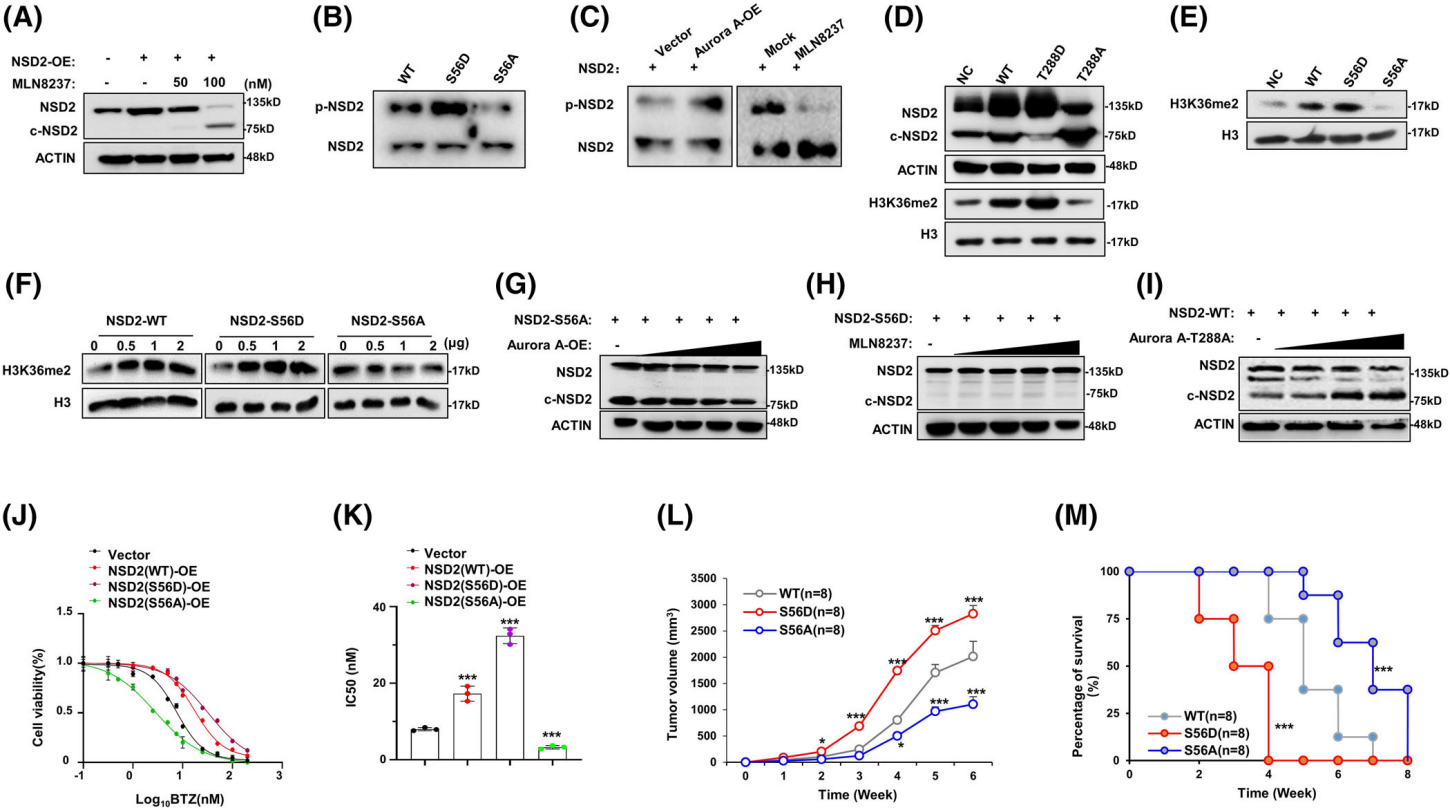
Aurora A phosphorylates NSD2 at S56 residue to enhance NSD2 activity. (A) Representative western blotting (*n* = 3) showing NSD2 cleavage in LP‐1 cells infected with virus packing pLV‐NSD2(WT)‐FLAG for 48 h while treating with gradient concentration of MLN8237 for 6 h. (B) Representative western blotting (*n* = 3) showing phospho‐NSD2 level with purified NSD2 (WT/S56A/S56D) shifted by Mn^2+^‐Phospho‐tag SDS‐PAGE gel. (C) Representative western blotting (*n* = 3) showing phospho‐NSD2 level with purified NSD2 shifted by Mn^2+^‐Phospho‐tag SDS‐PAGE gel accomplished by Aurora A‐OE or MLN8237 treatment. (D) Representative western blotting (*n* = 3) showing NSD2 and H3K36me2 in LP‐1 cells infected with virus packing wild type (WT), or mutation of Aurora A (T288D or T288A). (E) Representative western blotting (*n* = 3) showing H3K36me2 level in LP‐1 cells infected by virus packing wild type (WT) or NSD2 mutations (S56A or S56D). (F) Representative western blotting (*n* = 3) showing H3K36me2 level in LP‐1 cells infected with increasing ratios of virus packing pLV‐NSD2‐Flag (WT/S56A/S56D). (G) Representative western blotting (*n* = 3) showing NSD2 cleavage level in LP‐1 cells infected with virus packing pLV‐NSD2 (S56A)‐FLAG and increasing ratios of virus packing pITA‐AURKA‐HA for 48 h, (H) packing pLV‐NSD2(S56D)‐FLAG in the presence of gradient concentration of MLN8237 for 48 h and (I) pLV‐NSD2(WT)‐FLAG and increasing ratios of virus packing pITA‐AURKA(T288A)‐HA. (J) Alternation of IC50 to BTZ in LP‐1 cells infected with virus packing pLV‐NSD2‐Flag (WT/S56A/S56D), and (K) showing the statistical analysis of IC50 detected by CCK8 (*n* = 3). (L) Tumour growth of NSD2(S56A)‐LP‐1, NSD2(S56D)‐LP‐1 and NSD2(WT)‐LP‐1 cells derived tumours in NSG mice treated with BTZ (0.5 mg/kg, i.p.). Tumour volume = 1/2(L*W^2^) mm, where the L presenting the length and W representing width of tumour. (M) Survival rate of mice bearing NSD2(S56A)‐LP‐1, NSD2(S56D)‐LP‐1 and NSD2(WT)‐LP‐1 cells treated with BTZ (0.5 mg/kg, i.p.) when tumour achieves 15 mm. *, *p* < .05; *** *p* < .001

**FIGURE 5:**
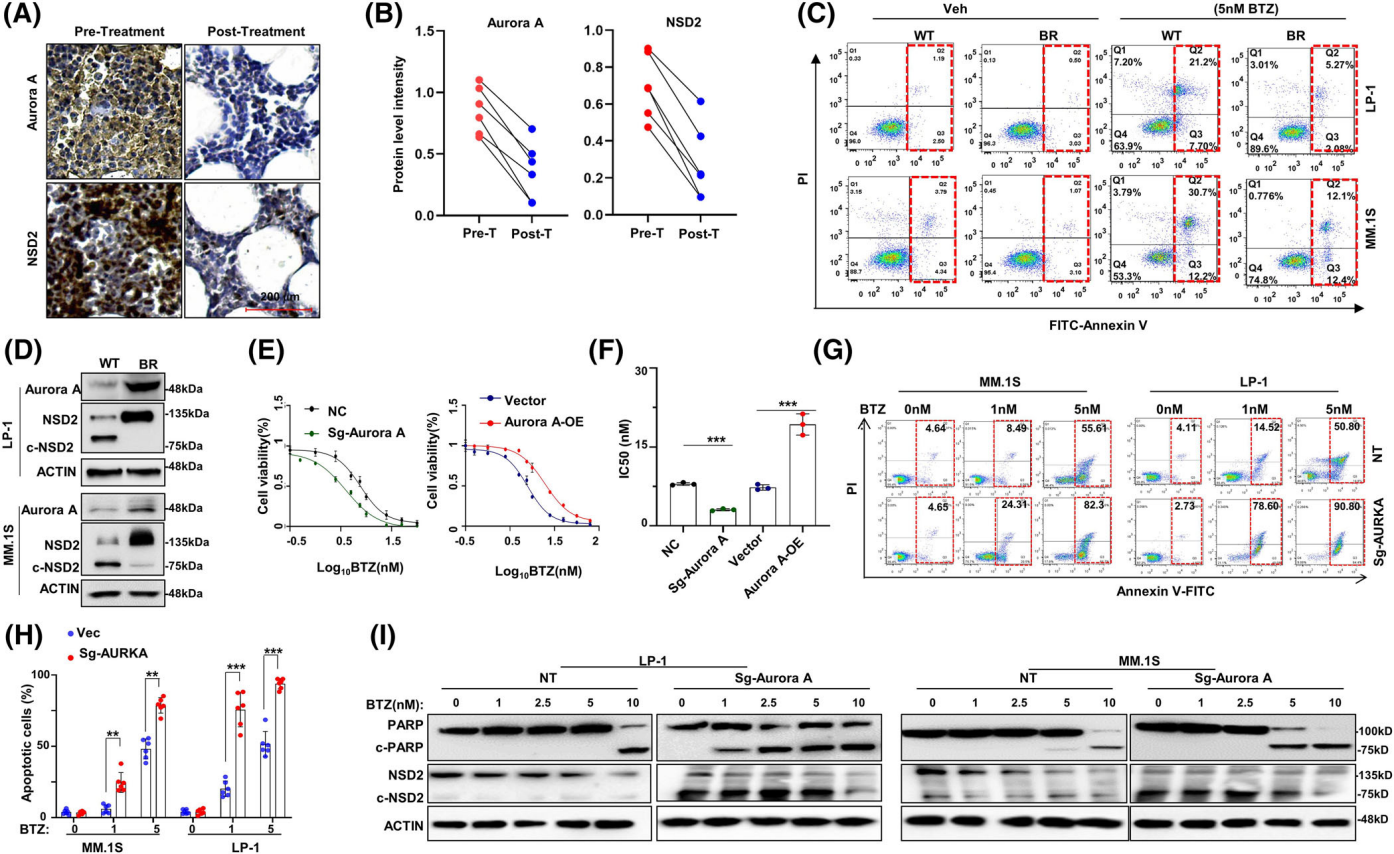
Aurora A alters the sensitivity of multiple myeloma (MM) cells to bortezomib (BTZ). (A) Representative immunohistochemical staining (*n* = 6) of Aurora A and NSD2 protein in the bone marrow slides from a MM patient acquired complete response (CR), and (B) the statistical analysis of NSD2 and Aurora A protein levels before (pre‐T) and after (post‐T) treatment in six patients with CR. (C) Representative flow cytometry assay (n = 3) of apoptotic cells in BR LP‐1 and MM.1S cells induced with BTZ (5 nM) for 48 h. (D) Representative western blotting (*n* = 3) showing the alternation of NSD2 and Aurora A protein levels in LP‐1 and MM.1S BR cells compared with the wild‐type (WT). (E) The alternation of IC50 to BTZ in Aurora A‐KO and Aurora A‐OE LP‐1 cells, and (F) statistical analysis of IC50 (*n* = 3). (G) Representative flow cytometry showing apoptotic cells in Aurora A‐KO LP‐1 cells induced by increasing concentration of BTZ, and (H) showing statistical analysis of flow cytometry (*n* = 6). (I) Representative western blotting (*n* = 3) showing NSD2 and cleavage of PARP induced by gradient concentration of BTZ in Aurora A‐KO LP‐1 and MM.1S cells compared with non‐target control. ** *p* < .01; *** *p* < .001

To further determine how Aurora A protects NSD2, we ectopically expressed the NSD2‐flag protein in HEK‐293T cells and then treated with MLN8237, and notified a 75 kDa truncation of NSD2 was cleaved at a dose‐dependent manner (Figure 4A). Indeed, through mass spectrum assay, we found that Aurora kinase A could phosphorylate NSD2 at S56 residue (Figure S4A). To validate a direct phosphorylation, we performed phospho‐tag SDS‐PAGE electrophoresis, and indeed observed that active mutation at S56 (S56D) augmented, but inactivate mutation (S56A) abolished the phosphorylation of NSD2 (Figure 4B), which could mimic the similar effect of gain‐of‐function of Aurora A through ectopic expression, or pharmacologically loss‐of‐function of Aurora A using MLN8237 (Figure 4C). Furthermore, when the threonine 288 of Aurora A was mutated to aspartic acid (T288D), a mutation of persistent activation, or to arginine (T288A), a mutation abolishing phosphorylating activation, NSD2 protein levels could be accordingly upregulated or suppressed, along with the histone methyltransferase activity of NSD2 augmented or inhibited, respectively (Figure 4D). Since Aurora A phosphorylated NSD2 protein at S56, we assessed the function of NSD2 with S56 mutated to aspartic acid (S56D) or to arginine (S56A), and found that S56D‐NSD2 showed an increasing methyltransferase function reflected by even higher H3K36me2 level, however, S56A‐NSD2 resulted in an even lower H3K36me2 level compared with WT NSD2, respectively (Figure 4E), and all these effects were in a dose‐dependent manner (Figure 4F). Furthermore, Aurora A could not protect S56A‐NSD2 from cleavage (Figure 4G), while S56D‐NSD2 could not be cleaved by pharmacological inhibition of Aurora A (Figure 4H), and the loss of protection was also observed in NSD2 degradation (Figure S4B,C). In addition, the inactive mutation of Aurora A (T288A) augmented the truncation of NSD2 (Figure 4I). Significantly, S56D‐NSD2 overexpressing MM cells rendered resistance to BTZ while the S56A‐NSD2 drove MM cells more sensitive to BTZ treatment (Figure 4J,K), which were similar with the effects of T288D‐Aurora A and T288A‐Aurora A in MM cells, respectively (Figure S4D). Notably, neither pharmacologically inhibition of Aurora A (Figure S4E) nor mutations of Aurora A enzyme activity residue (Figure S4F) and NSD2 phosphorylation residue (Figure S4G) altered the protein‐protein interaction. In vivo, BTZ exerted limited inhibitory effect on NSD2(S56D)‐LP‐1 cells derived tumours, but exhibited marvellous anti‐MM growth effect on the NSD2(S56A)‐LP‐1 cells derived tumours, when compared to the wild type NSD2 (NSD2(WT)‐LP‐1) derived tumours (Figure 4L), and the overall survival was significantly improved only in mice bearing NSD2(S56A)‐LP‐1 cells derived tumours (*p *< .001) (Figure 4M). Collectively, all the above findings demonstrate that Aurora A induces resistance to BTZ by stabilizing the NSD2 protein and enhancing methyltransferase activity through directly phosphorylating NSD2 at S56 residue. Intriguingly, clinical samples from patients acquired complete response after BTZ‐based regimen treatment showed protein levels of Aurora A and NSD2 were accordantly declined (Figure 5A), and these two protein levels were all significantly declined in six patients with complete response (Figure 5B). Moreover, in BR MM cells that are non‐responsible to BTZ treatment (Figure 5C), NSD2 and Aurora A both highly augmented despite of cytogenetic background (Figure 5D). As expected, Aurora A overexpression induced MM cells more resistance to BTZ, and depletion or pharmacologically inhibition of Aurora A drove MM cells more sensitive to BTZ (Figure 5E,F and Figure S5A). Moreover, overexpression of Aurora A failed to decrease the anti‐MM effect of BTZ on NSD2‐KO MM cells (Figure S5B). Similar results were also observed in another proteasome inhibitor Carfilzomib, but not in melphalan (Figure S5C,D). Meanwhile, depletion of Aurora A sensitizing MM cells to BTZ treatment was also evidenced by more apoptosis through flow cytometry assay (Figure 5G,H), and more cleaved PARP following suppressed NSD2 level according to gradient BTZ concentration (Figure 5I). Notably, these effects were all more significant in LP‐1 cells with t(4;14) than MM.1S cells without t(4;14).

### 3.4 Aurora A cooperates with NSD2 to regulate chemoresistance genes in MM cells

**FIGURE 6:**
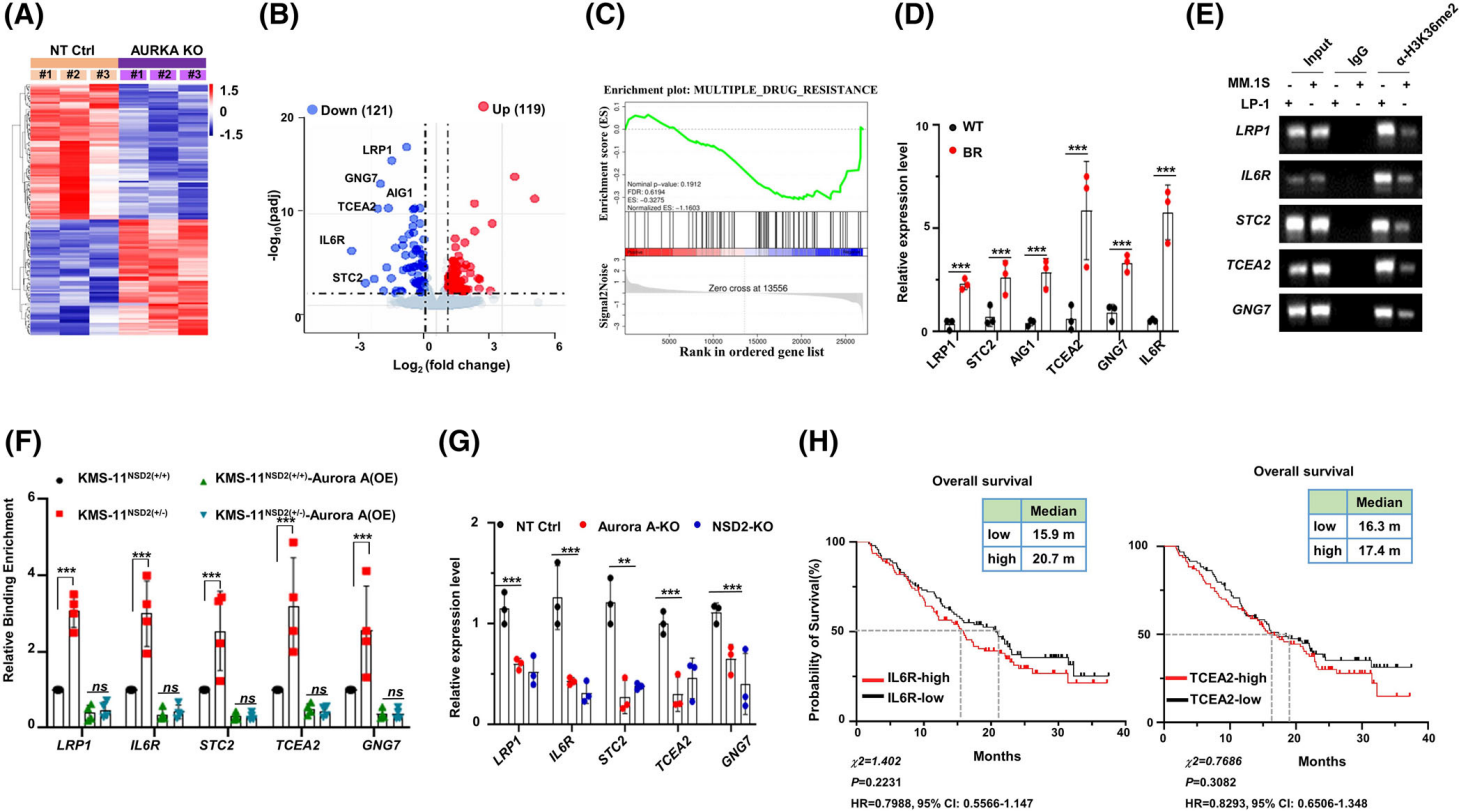
RNA sequencing reveals that NSD2 and Aurora A alter the transcriptome of multiple myeloma (MM) cells to adapt drug resistance. (A) Heat map of differently expressed genes in Aurora A‐KO LP‐1 cells by bulk RNA sequencing. Gene expression is shown in normalized log2 counts per million. (B) Volcano plot showing differentially expressed genes in Aurora A‐KO LP‐1 cells. Number of genes downregulated or upregulated in Aurora A‐KO LP‐1 cells is shown on the top (fold change > 1.2; adjusted *p* value < .05). (C) GSEA analysis shows genes enrichment in multiple drug resistance pathway in Aurora A‐KO sample. (D) Quantitative polymerase chain reaction (qPCR) validating the target genes in bortezomib‐resistant (BR) and wild‐type (WT) LP‐1 cells. (E) ChIP‐PCR results showing binding of promoters of *LRP1*, *IL6R*, *STC2*, *TCEA2* and *GNG7* in LP‐1 and MM.1S cells using anti‐H3K36me2 and normal rabbit IgG. (F) ChIP‐qPCR results showing the relative enrichment abundance of H3K36me2 on promoters of *LRP1*, *IL6R*, *STC2*, *TCEA2* and *GNG7* genes in NSD2‐KO‐KMS‐11 cells. (G) qPCR validating the target genes in Aurora A‐KO and NSD2‐KO LP‐1 cells. (H) The prognostic value of IL6R (left) and TCEA2 (right) in the overall survival curve in database GSE9782 (cutoff, median). ** *p* < .01; *** *p* < .001

In order to identify genes that are coordinately regulated by Aurora A and responsible to drug resistance, we applied bulk RNA sequencing in LP‐1 cells with or without AURKA depletion to screen differentially expressed genes and ChIP‐PCR assay using the H3K36me2 antibody to assess whether expressions of these genes were directly regulated by H3K36me2. Differential gene expression analysis revealed 121 genes significantly downregulated and 119 genes significantly upregulated in the Aurora A depletion cells (Figure 6A,B). KEGG analysis highlighted that downregulated genes were involved in signal transduction, metabolism of cofactors and vitamins, transport and catabolism, drug resistance and cell death, which may promote cancer progression and responsible for treatment tolerance (Figure S6A). GO analysis also highlighted cell adhesion and apoptosis alternation in the Aurora A‐KO LP‐1 cells (Figure S6B). In addition, the GSEA enrichment scores were negatively correlated with drug resistance pathway (Figure 6C), ABC family transporters and IL6 signalling pathway due to Aurora A‐KO (Figure S6C). It is a remarkable fact that some highly decreased genes, such as *IL6R, ERBB3, LRP1, STC2, SULF2, GAA, ZNF467, SLC37A1, AIG1, TCEA2, TXNDC5* and *GNG7* were cancer drug–resistance related, of which qPCR results validated that *LRP1, STC2, AIG1, TCEA2, GNG7* and *IL6R* all augmented in the BR MM cells (Figure 6D). We found promoter regions of *LRP1, STC2, TCEA2, GNG7* and *IL6R* genes could be enriched by H3K36me2 antibody, and enrichment abundance was extraordinarily higher in LP‐1 cells (Figure 6E). Using commercially established NSD2‐KO KMS‐11 cells, we found that Aurora A could only augment the binding enrichment in ^(NSD2+/+)^KMS‐11 cells, but failed to affect the binding enrichment in ^(NSD2+/‐)^KMS‐11 cells (Figure 6F). Interestingly, expressions of these genes were all downregulated both in Aurora A depletion and in NSD2 depletion MM cells (Figure 6G). When Aurora A was overexpressed in the NSD2‐KO LP‐1 cells, expressions of these genes were barely improved (Figure S6D); however, when NSD2 was overexpressed in the Aurora A‐KO LP‐1 cells, the gene expressions were remarkably rescued (Figure S6E).These data strongly suggested that these genes were regulated by Aurora A‐NSD2 axis. Of note, analysis of clinical cohort GSE9782 by median value of expression showed that higher expressions of *IL6R* and *TCEA2* predicted poorer trends in prognosis of MM patients (Figure 6H), and optimised expression values of *IL6R, STC2* and *TCEA2* expressions predicted significant negative correlations with overall survival in the cohort GSE4581 (Figure S7A). Functionally, depletion of *IL6R, STC2* and *TCEA2* all drove MM cells more sensitive to BTZ treatment (Figure S7B,C). In line with it, Aurora A was higher in CD138^low^ plasma cells from bone marrow compared with CD138^high^ plasma cells (Figure S7D), while previous study has confirmed that MM patients rendering lower CD138 predicted poorer prognosis.^30^ These results further support a crucial role of Aurora A and NSD2 regulating loop in inducing MM BTZ resistance and poor prognosis.

### 3.5 Aurora A inhibitor sensitizes t(4:14) MM cells to BTZ in vivo

**FIGURE 7:**
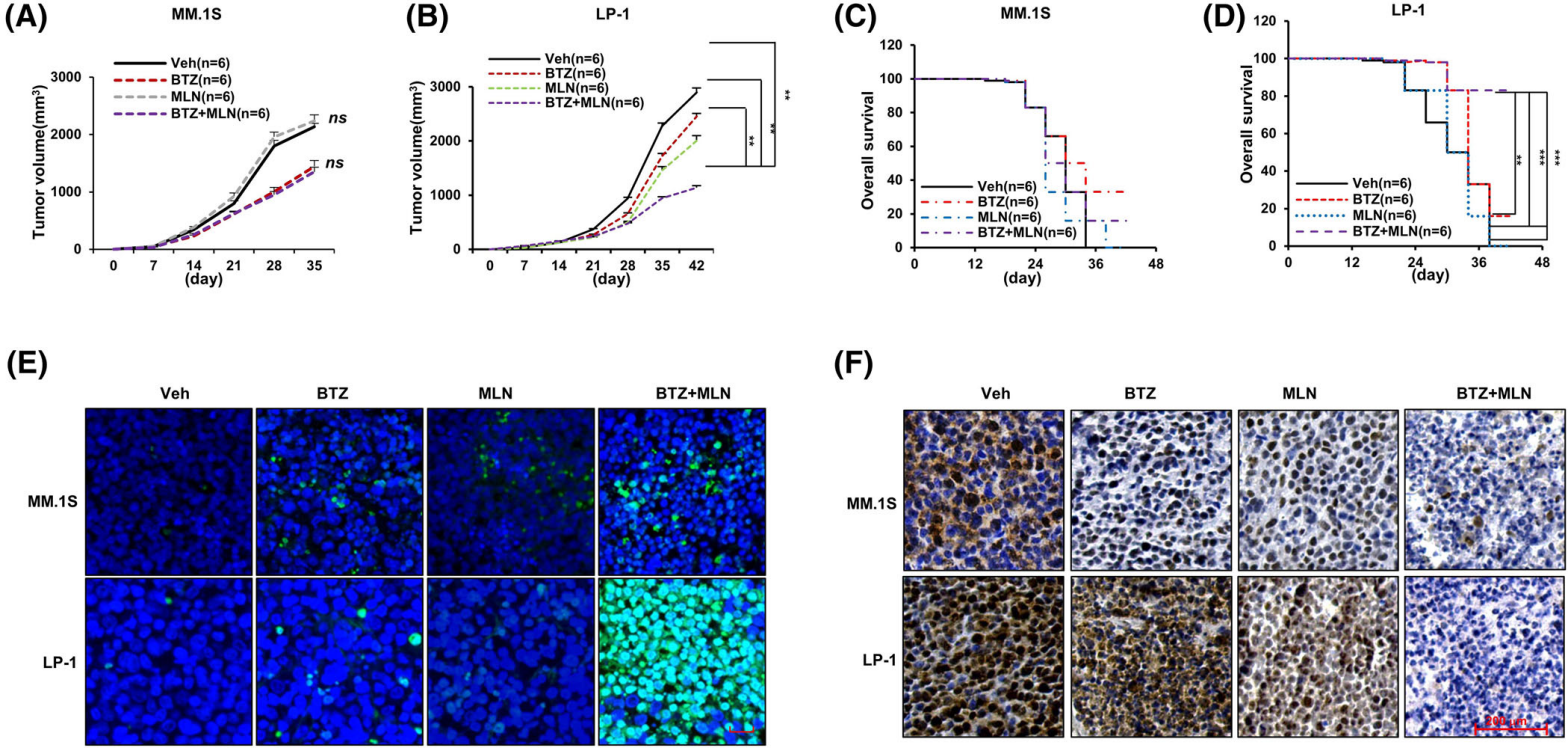
Synergistic anti‐tumour growth effect of MLN8237 with bortezomib (BTZ) in xenograft model. (A) Tumour growth of MM.1S cells generated xenograft and (B) LP‐1 cells originated xenograft in mice treated with BTZ (0.5 mg/kg, i.p.) with or without MLN8237 (5 mg/kg, with β‐cyclodextrin, i.g.). Tumour volume = 1/2(L*W^2^) mm, where the L presenting the length and W representing width of tumour. (C) Survival rate of mice bearing MM.1S or (D) bearing LP‐1 cells in the above groups when tumour achieves 15 mm. **, *p* < .01; ***, *p* < .001. (E) TUNEL assay showing apoptotic cells in tissues from MM.1S and LP‐1 cells derived xenografts (*n* = 3). Scale bar, 200 μm. (F) Representative immunohistochemical staining of NSD2 protein in the tissues from xenograft models treated with BTZ or BTZ plus MLN8237. Scale bar, 200 μm. ** *p* < .01; *** *p* < .001

**FIGURE 8:**
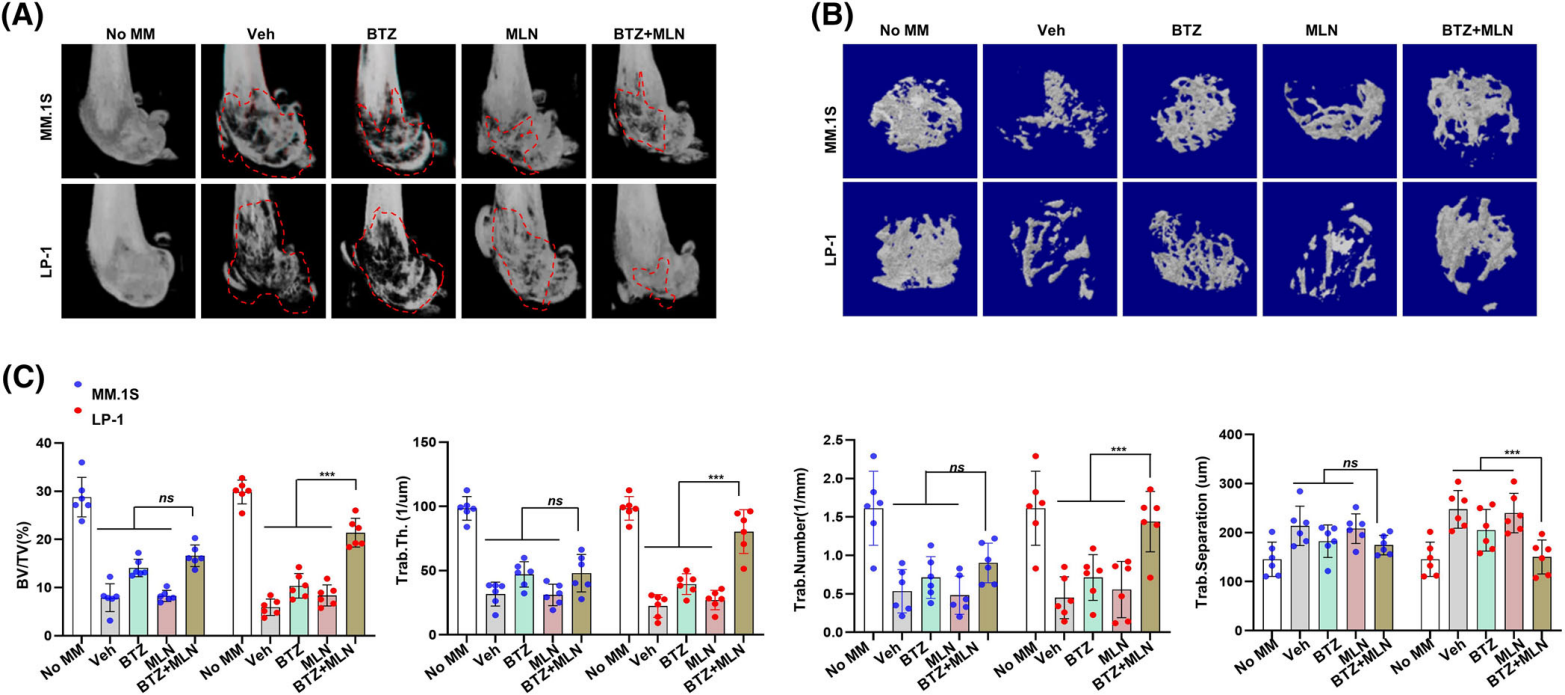
Synergistic effect of MLN8237 and bortezomib (BTZ) in alleviating bone lesion in mice. (A) Representative microCT reconstructions of mouse femurs bearing MM.1S and LP‐1 cells (1 × 10^6^/mouse) and treated with BTZ (0.5 mg/kg) (*n* = 6) or BTZ plus MLN8237 (*n* = 6), and (B) 3D reconstructions of bone trabecula in metaphyseal region. (C) Measurement of the percentage of bone volume to total volume (BV/TV), the cortical thickness, number of bone trabecula and trabecula separation in the metaphyseal region of the mice femur in the BTZ or BTZ plus MLN8237 groups. *** *p* <.001. Data are averages ± SD (*n *= 6 mice/group)

Finally, to evaluate the therapeutic relevance of our findings, we constructed a xenograft model using NOD/SCID mice and administered with two agents, BTZ and MLN8237, alone or together. Combination of BTZ and MLN8237 exerted limited synergistic effect on MM.1S cells derived tumours, but a marvellous synergistic anti‐MM growth effect was observed on LP‐1 cells derived xenograft model (Figure 7A,B), and overall survival was significantly prolonged only in mice bearing LP‐1 cells derived tumours (Figure 7C,D). Meanwhile, number of apoptotic cells was significantly augmented in the LP‐1 cells derived tissues compared to MM.1S derived tissues (Figure 7E), and NSD2 level was significantly reduced in collaterally treated groups (Figure 7F). In another bone lesion model constructed by injecting MM cells into the femur of NOD/SCID mice, significant remission of the bone lesion was found in the LP‐1 cells bearing mice compared with MM.1S cells bearing mice in the combination‐treated group (Figure 8A). 3D reconstruction of the bone trabecula measurements also showed a better trabecular network at the metaphyseal regions of LP‐1 cells bearing mice femurs in the combination treatment group (Figure 8B). Furthermore, quantitative analysis showed significant recovery of bone destruction in the LP‐1 cells bearing mice femurs in the combination treatment group, as evidenced by higher percentages of BV/TV, cortical thickness, more trabecula numbers and lower size of trabecula separation at the metaphysis and the diaphysis (Figure 8C). Taken together, these data demonstrate that MLN8237 sensitizes the anti‐MM effect of BTZ in vivo.

## 4 DISCUSSION

In the current study, we report that Aurora A inhibitor shows synergistic effect with BTZ on t(4;14) positive MM cells, and reveal a previously not discovered positive regulating loop between phosphorylation of NSD2 and methylation of Aurora A. Thus, the t(4;14) translocation rendering high level of NSD2 could be used as a hallmark for accessing application of MLN8237 with BTZ based therapy regimen in this subtype of MM patients in order to achieve higher response rate and overcome relapse and refractory.

BTZ is still the first‐line therapy for management of MM in the clinic,^31^ while the intrinsic or acquired chemoresistance is a major obstacle of MM therapy. So far, combinations of PIs and other drugs may be effective approaches. Aurora A belongs to a highly conservative serine‐threonine kinases family and is essential to tumorigenesis and progression, mainly by controlling cell cycle at G1/S phage.^32^ However, a growing body of evidences have identified that excessive Aurora A appeared in all cell cycle stages, and also presented in cytoplasm of tumour cells rather than only in centrosome of normal cells, indicating that Aurora A is closely correlated with tumorigenesis.^33^ In our study, we identified an Aurora A specific inhibitor, MLN8237, from an epigenetics compound library, as one of the most effective compounds possessing synergetic anti‐MM effect with BTZ. Interestingly, the synergetic effect was only observed in the t(4;14) positive MM cells. It is well known that t(4;14) is closely correlated with MM progression,^34^ but its roles in treatment response have not been well elucidated. We discovered a previously unknown phenotype that Aurora A and NSD2 protein levels were positively correlated in t(4;14) positive MM cell lines, as well as samples from MM patients, and these two molecules orchestrated MM cells resistance to proteasome inhibitors.

Posttranslational modifications, such as phosphorylation and ubiquitination, play crucial roles in modulating protein stabilization and function.^35^, ^36^ It has been well known that NSD2 promotes MM progression through histone methyltransferase activity,^34^, ^37^ while regulation of NSD2 protein itself is not well investigated. We hypothesized that Aurora A may phosphorylate NSD2 to alter protein stability and function. As to the mechanism of Aurora A overexpression in t(4:14) positive MM cells, we speculate that Aurora A level is regulated mainly at posttranslational level by NSD2, but we can't exclude other factors affecting overexpression of Aurora A. By immunoprecipitation of NSD2 protein and mass spectrum assay, we identified that the direct interaction between Aurora A and NSD2 could achieve the phosphorylation of NSD2 at serine 56 residue, and consequently improve its methyltransferase activity. Our data also indicated that phosphorylation of NSD2 at S56 suppressed both the degradation and cleavage of NSD2, which are critical for NSD2 protein stability and enzyme activity.^38^, ^39^ We identified that Aurora A‐mediated serine 56 phosphorylation of NSD2 plays critical role in cleavage and ubiquitination of NSD2, mainly through K11‐ and K29‐linked ubiquitin chains, in which K11‐linked ubiquitination leads to protein degradation,^40^ and K29‐linked ubiquitination is responsible for protein‐protein interaction and degradation.^41^, ^42^ However, we couldn't define the causal consequence of cleavage and ubiquitination in the current study, which need further investigations. Notably, this bidirectional regulation between Aurora A and NSD2 seems independent of cell cycle. In addition, we identified a series of genes known as drug resistance‐related regulators, such as *IL6R, STC2* and *TCEA2*, whose expressions were synchronously suppressed by *NSD2* and *AURKA* depletion. A number of studies conclude that Aurora A phosphorylates proteins both in cytoplasm and nuclei in the entire cell cycle process.^33^, ^43^ For example, Aurora kinase family could be a potential target of MM treatment for the interaction with the key regulators of canonical and non‐canonical NF‐κB pathways,^19^ and the latter is a well acknowledged key mediator of MM carcinogenesis and drug resistance.^20^ NSD2 is also implicated in cancer progression via methylation of Aurora A, but our study is the first report of NSD2 as the downstream target of the Aurora A. A recent study also showed NSD2 could be phosphorylated by AKT pathway to promote its stability, which is consistent with our conclusion.^44^ Thus, our results revealed an important previously unknown positive regulating loop between NSD2 and Aurora A in promoting MM progression.

Our study also provides rationale for adopting new inhibitors for overcoming refractory or relapsed MM patients. Previous study indicates that MLN8237 alone could induce anti‐proliferation and apoptosis of MM cells at a high dose manner,^22^ and a phase 1 clinical trial study showed MLN8237 had antitumour activity as a single agent in MM patients.^24^ However, a phase II clinical trial of MLN8237 showed unacceptable toxicity on myelosuppression, therefore it was not recommended for further study as a treatment for MM.^23^, ^24^ Thus, the current study clarifies that Aurora A selective inhibitor probably is more suitable for MM patients with t(4;14) genetic background due to its property of undermining NSD2 stability and activity.

## 5 CONCLUSIONS

The current study uncovers a novel reciprocal regulation between Aurora A and NSD2 in promoting MM sensitivity to PIs, in which, Aurora A can stabilize NSD2 via phosphorylating NSD2 at S56 residue to promote MM chemoresistance. The clinical significance of our study is to provide evidence for applying MLN8237 with PIs to augment response in MM patients with t(4;14) abnormality. In addition, when using Aurora A inhibitor or other drugs as a combination treatment strategy, heterogeneity should be taken into consideration to achieve better outcomes, and to benefit the personalised management in the clinic.

## ETHICS APPROVAL AND CONSENT TO PARTICIPATE

This study was approved by the Ethics Committee of Tianjin Medical University and all protocols conformed to the Ethical Guidelines of the World Medical Association Declaration of Helsinki. Signed informed consent was obtained from each participating individual prior to participation in the study. Animal studies were approved by the Committee on Animal Research and Ethics of Tianjin Medical University and all protocols conformed to the Guidelines for Ethical Conduct in the Care and Use of Nonhuman Animals in Research.

## CONFLICT OF INTERESTS

The authors declare no potential conflicts of interest.

## Supporting information



SUPPORTING INFORMATION

SUPPORTING INFORMATION

SUPPORTING INFORMATION

SUPPORTING INFORMATION
