# Supplementary material for: Posttranslational modification of Aurora A‐NSD2 loop contributes to drug resistance in t(4;14) multiple myeloma
Source: Clin Transl Med. 2022 Apr 7;12(4):e744. doi: 10.1002/ctm2.744 (PMC8989081; doi:10.1002/ctm2.744)
Supplement: Supplementary file 1 — SUPPORTING INFORMATION [file CTM2-12-e744-s001.docx]

**Supplementary Figures**

**
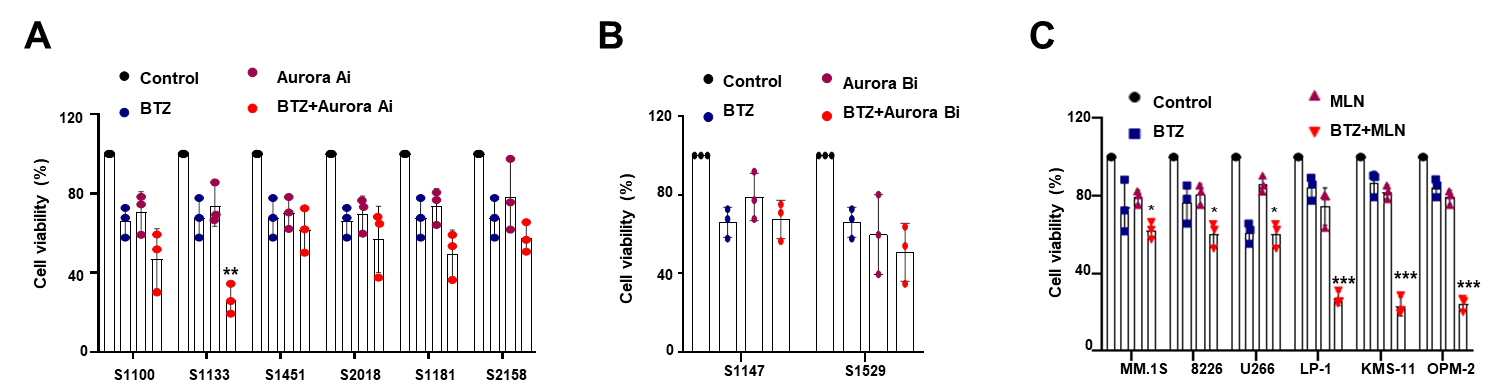
**

**S Figure 1 Synergistic anti-MM effect of Aurora A inhibitor with BTZ on t(4;14) MM cells.**

Cell viability was determined in LP-1 cells treated with other six Aurora A inhibitors (A) and two Aurora kinase B inhibitors (B) using CCK8 kit. (C) The cell viability of 8226, MM.1S, U266, LP-1, KMS-11 and OPM-2 cells treated with 1nM BTZ and MLN8237 (25×BTZ dosage) for 48 hours. ** *P* < 0.01, *** *P* < 0.001. All data represent at least three independent experiments.


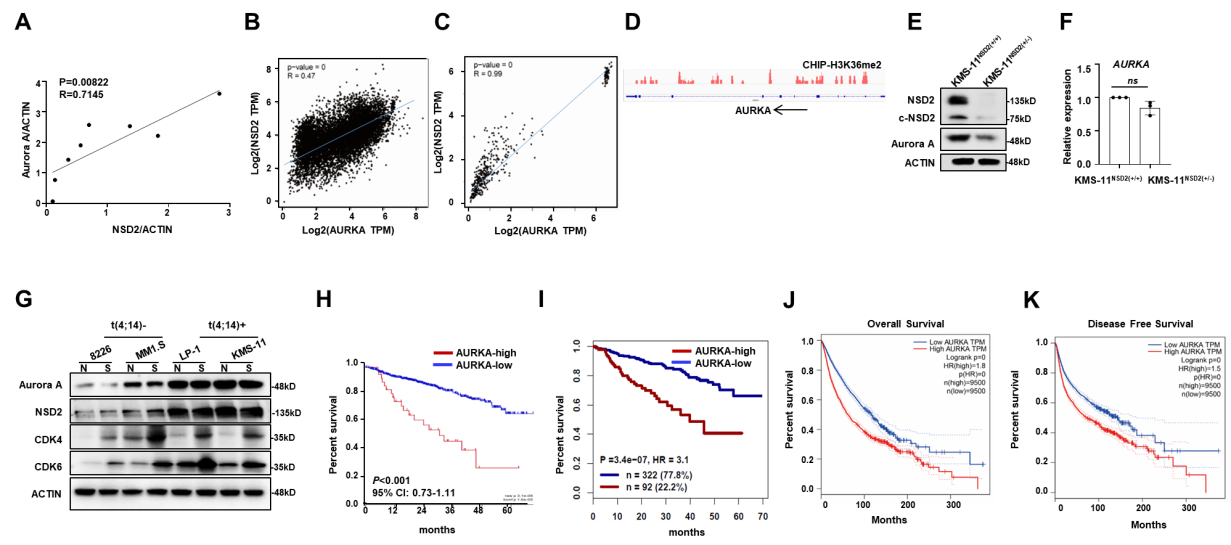


**S Figure 2 Correlation of Aurora A and NSD2 in t(4;14) MM cells and the clinical significance.**

(A) Pearson correlation analysis of NSD2 and Aurora A protein levels in MM cells. The correlation between AURKA and NSD2 in all tissues (B) and only in blood samples (C) (GEPIA). (D) Gene tracks of representative ChIP-seq profiles for the H3K36m2 mark at the *AURKA* gene loci. (E) Representative images of western blotting (n=3) showing NSD2 and Aurora A protein levels in KMS1-11^(NSD2+/+)^ and KMS1-11^(NSD2+/-)^ cells. (F) mRNA level of *AURKA* in KMS1-11^(NSD2+/+)^ and KMS1-11^(NSD2+/-)^ cells. (G) Representative images of western blotting (n=3) showing NSD2 and Aurora A protein levels in 8226, MM.1S, LP-1 and KMS-11 cells with starvation for 12 hours compared with normal condition. N, normal condition; S, starvation. (H) The prognostic value of AURKA in the overall survival curve (GSE2658) in MM patient (cutoff, optimize expression values). (I) The prognostic value of AURKA in the overall survival curve (GSE4581) in MM patient (cutoff, optimize expression values). The prognostic value of AURKA in the overall survival curve (J) and the disease free survival curve (K) (GEPIA) in blood disease.

**
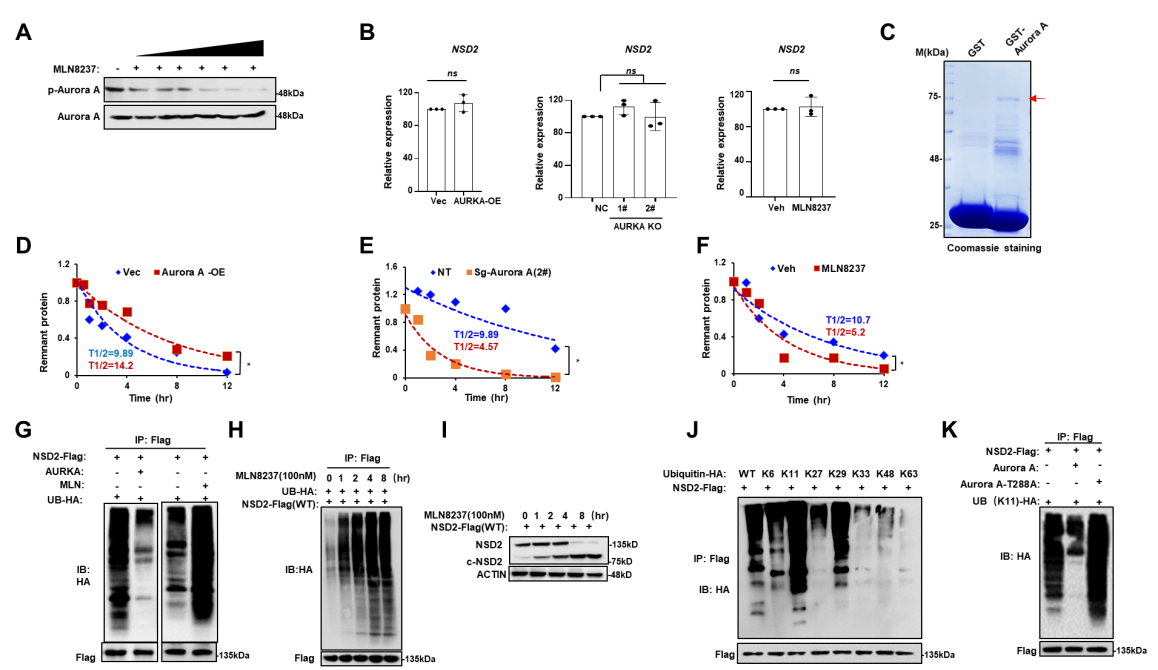
**

**S Figure 3 Aurora A stabilizes NSD2 protein from degradation by ubiquitination.**

(**A**) Representative western blotting qualified the effect of MLN8237 on Aurora kinase A in LP-1 cells. (**B**) mRNA level of *NSD2* in AURKA-OE, AURKA-KO LP-1 and MLN8237 treated LP-1 cells. (C) The SDS-PAGE gel was stained by Coomassie brilliant blue to verify GST-Aurora A fusion protein. Densitometric analysis of immunoblots of degradation rate of NSD2 protein from three independent experiments was used to calculate the half-life of NSD2 in Aurora A-OE LP-1(D), Aurora A-KO LP-1(E) and MLN8237 treatment LP-1(F) cells treated with 20 μM cycloheximide (CHX) for up to 12 hours. * *P* < 0.05. All data represent at least three independent experiments. (G) Representative western blotting of the ubiquitination level of NSD2 due to Aurora A overexpression or MLN8237(100nM) treatment respectively. (H) Representative western blotting of the ubiquitination level of NSD2 in LP-1 cells due to increasing duration of MLN8237(100nM) treatment. (I) Representative western blotting (n=3) showing NSD2 cleavage in LP-1 cells due to increasing duration of MLN8237(100nM) treatment. (J) Representative western blotting of the different mutation of lysine-kinked ubiquitination level of NSD2, including K6, K11, K27, K29, K33, K48 and K63. (K) Representative western blotting of the K11-linked ubiquitination level of NSD2.

**
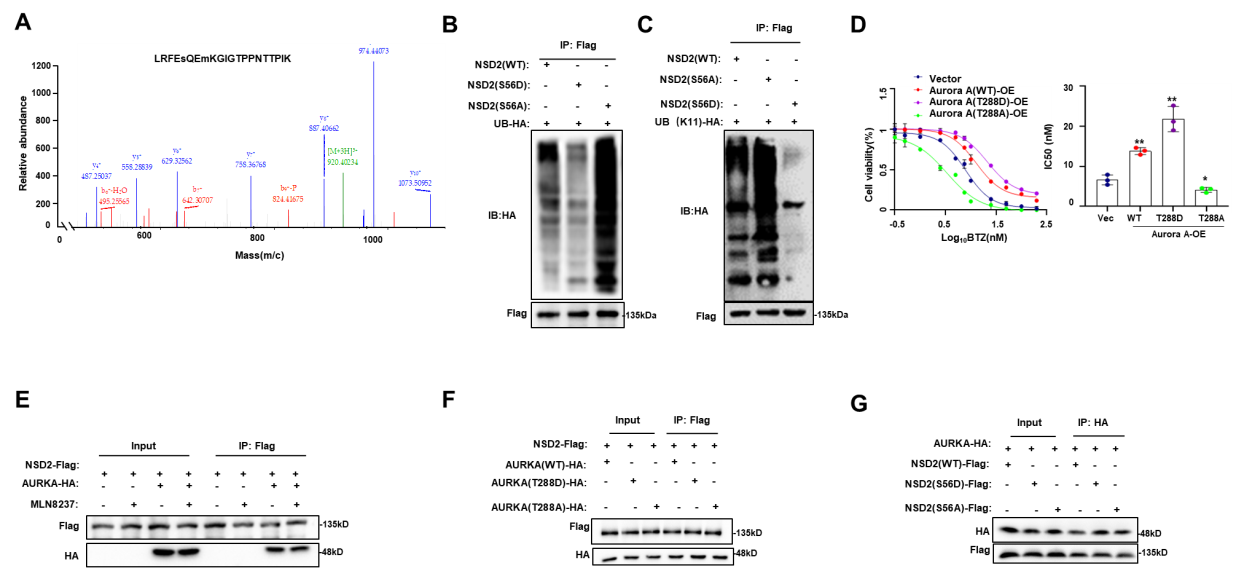
**

**S Figure 4 Aurora A phosphorylates NSD2 at S56 residue to protect NSD2.**

(A) Mass spectrum showing phosphorylation residue of NSD2 by Aurora A in HEK-293T cells transfected with pLV-NSD2-Flag and pITA-AURKA-HA for 48 hours. (B) Representative western blotting showed the ubiquitination level of wild type (WT) and mutated (S56A/S56D) NSD2. (C) Representative western blotting showed the K11-linked ubiquitination level of wild type (WT) and mutated (S56A/S56D) NSD2. (D) Alternation of IC50 to bortezomib in LP-1 cells infected with lentivirus carrying pITA-AURKA-Flag (WT/T288A/T288D), and the right panel showing statistical analysis of IC50 (n=3). (E) Representative images (n=3) of Co-IP showing exogenous interaction between Aurora A and NSD2 in HEK-293T cells transfected with pLV-NSD2-Flag and pITA-Aurora A-HA with or without MLN8237 treatment. (F) Representative images (n=3) of Co-IP showing exogenous interaction between Aurora A and NSD2 in HEK-293T cells. (G) Representative images (n=3) of Co-IP showing exogenous interaction between Aurora A and NSD2 in HEK-293T cells transfected with pLV-NSD2-Flag (WT/S56D/S56A) and pITA-Aurora A-HA.


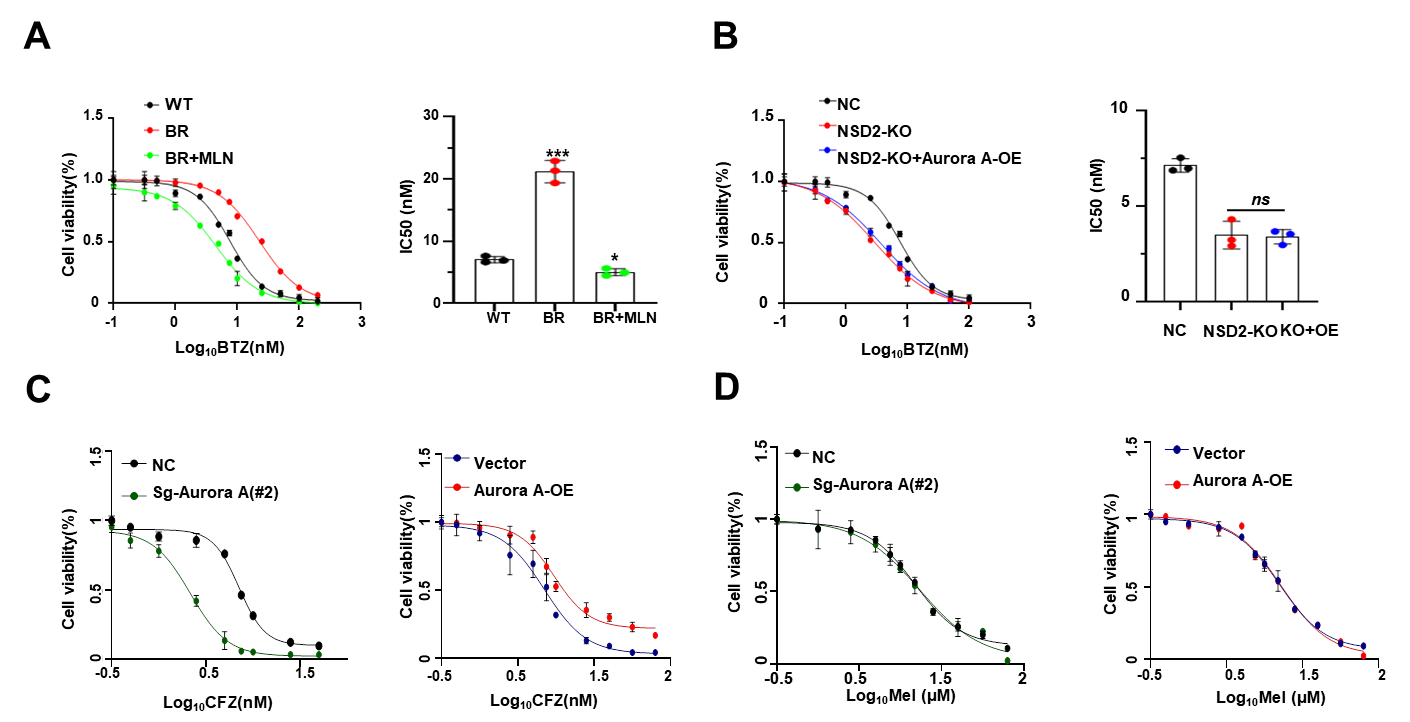


**S Figure 5 Aurora A alters the sensitivity of MM cells to BTZ.**

(A) The alternation of IC50 and statistical analysis of IC50 (n=3) to bortezomib in BR-LP-1 cells with or without MLN8237(100nM). (B) The alternation of IC50 and statistical analysis of IC50 (n=3) to bortezomib in NSD2-KO-LP-1 cells with or without Aurora A-OE. (C) Alternation of IC50 to carfilzomib in Aurora A-KO and Aurora A-OE LP-1 cells. (D) The alternation of IC50 to melphalan in Aurora A-KO and Aurora A-OE LP-1 cells.

**
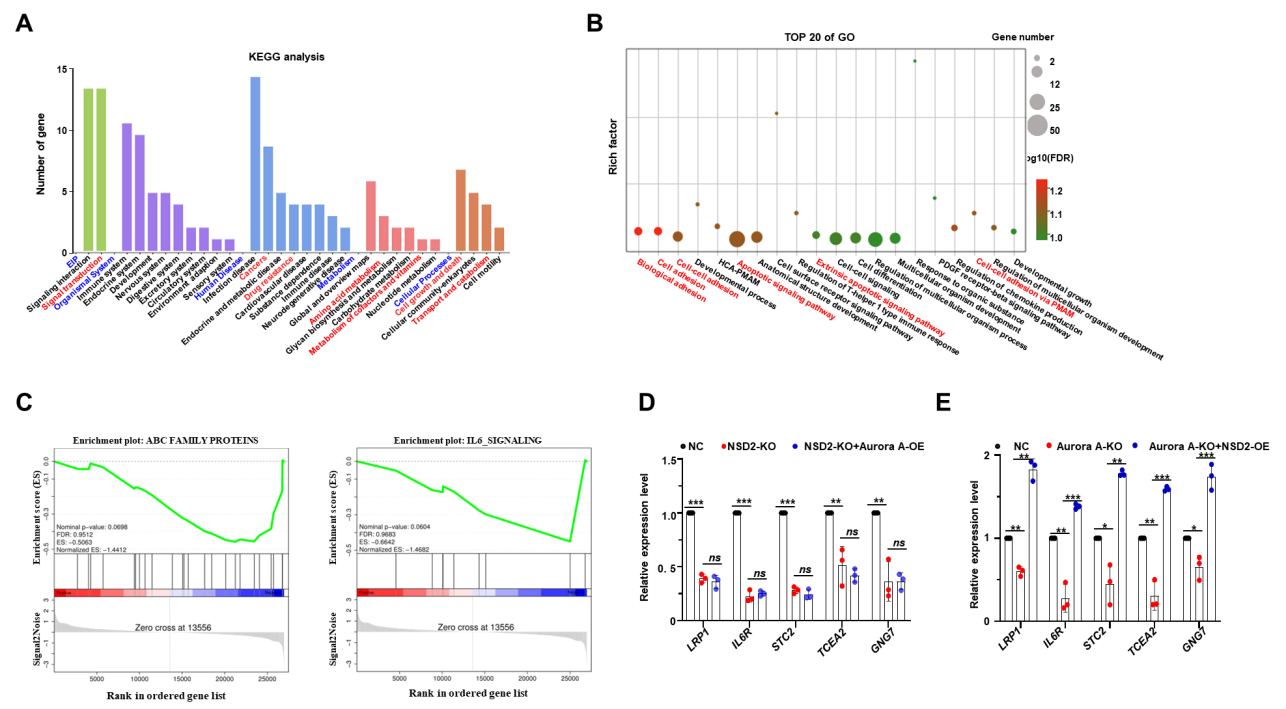
**

**S Figure 6 Transcriptome alteration of MM cells due to Aurora A-KO.**

(**A**) KEGG analysis showing the top five related pathway depleted biological processes in Aurora A-KO LP-1 cells. (**B**) Gene Ontology (GO) analysis showing top 20 biological processes in Aurora A-KO LP-1 cells. (C) GSEA analysis showing enrichments of genes associated with ABC family and IL6 signaling pathway in Aurora A-KO LP-1 cells. (D) qPCR validating the target genes in NSD2-KO and NSD2-KO + Aurora A-OE LP-1 cells. (E) qPCR validating the target genes in Aurora A-KO and Aurora A-KO + NSD2-OE LP-1 cells.


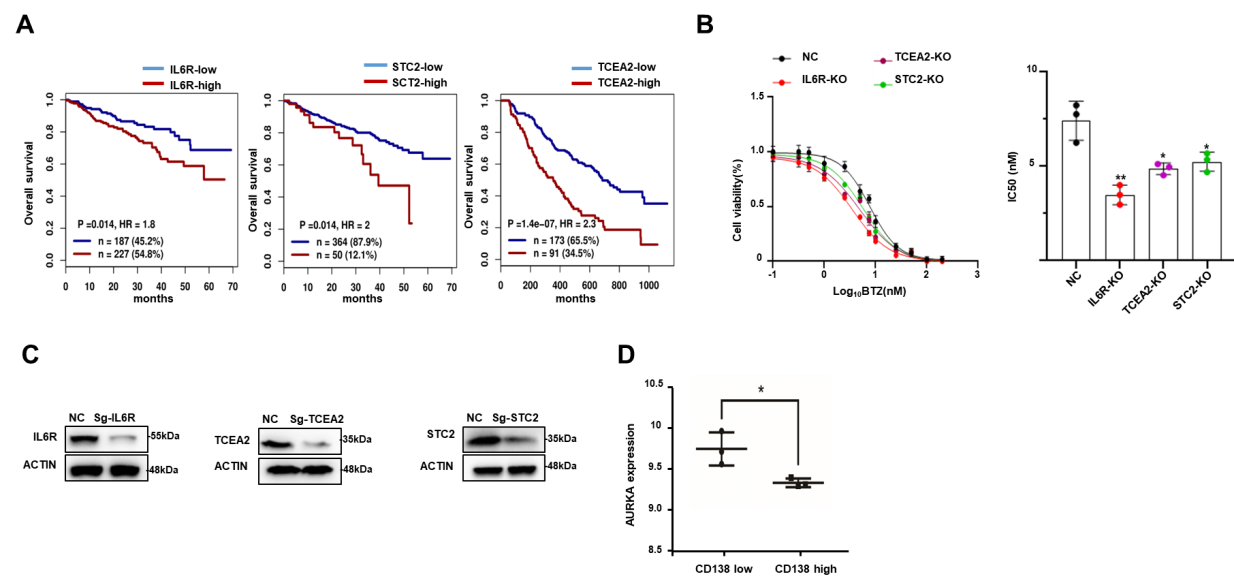


**S Figure 7 Functional analysis of Aurora A target genes in MM drug resistance.**

(**A**) The prognostic value of IL6R, STC2 and TCEA2 in the overall survival curve (GSE4581) in MM patient (cutoff, optimize expression values). (B) Alternation of IC50 to bortezomib in IL6R-KO, STC2-KO and TCEA2-KO LP-1 cells, and the statistical analysis of IC50 detected by CCK8 (n=3). (C) Representative western blotting qualified that IL6R-KO, STC2-KO and TCEA2-KO knock out effect in LP-1 cells. (**D**) The transcription level of AURKA between CD138-low and CD138-high cells in bone marrow.
