# Supplementary material for: Posttranslational modification of Aurora A‐NSD2 loop contributes to drug resistance in t(4;14) multiple myeloma
Source: Clin Transl Med. 2022 Apr 7;12(4):e744. doi: 10.1002/ctm2.744 (PMC8989081; doi:10.1002/ctm2.744)
Supplement: Supplementary file 3 — SUPPORTING INFORMATION [file CTM2-12-e744-s004.docx]

**Key resources of this study**

| **Category** | **Source** | **Cat. No.** |
| --- | --- | --- |
| **Antibodies** | | |
| Anti-rat Aurora A (D3E4Q) | Cell signaling technology | #14475 |
| Anti-HistoneH3(dimethylK36)antibody | Abcam | ab10799 |
| Anti-WHSC1/NSD2 antibody [29D1] | Abcam | ab75359 |
| Anti-rat HA-Tag (C29F4) | Cell signaling technology | #3724 |
| Anti-PARP | Cell signaling technology | 9532 |
| Anti-rat -H3 antibody | Abcam | [9662](https://www.cst-c.com.cn/products/primary-antibodies/caspase-3-antibody/9662?site-search-type=Products&N=4294956287&Ntt=caspase3&fromPage=plp) |
| Anti-mouse GAPDH | UTIBODY | UM4002 |
| Anti-IL6R | Proteintech | 23457-1-AP |
| Anti-TCEA2 | Cell signaling technology | #45010 |
| Anti-STC2 | Abcam | ab63057 |
| Anti-rabbit β-actin | Abclonal | AC006 |
| Goat Anti-Rabbit IgG-HRP | Sigma-Aldrich | A0545 |
| ANTI-FLAG® M2-Peroxidase | Sigma-Aldrich | A8592 |
| Rabbit Anti Mouse IgG-HRP | Sigma-Aldrich | A9044-2ML |
| Anti-rabbit IgG | Proteintech | 30000-0-AP |
| Anti-mouse IgG | Proteintech | B900620 |
| **Chemicals, Peptides and Recombinant Proteins** | | |
| 3FLAG peptide | Sigma-Aldrich | F4799 |
| FLAG Peptide | Sigma-Aldrich | F3290 |
| Human IL-6 standard | R&D system | 840245 |
| **Drugs** | | |
| Bortezomib (PS-341) | SelleckChem | S1013 |
| Epigenetics Compound Library | SelleckChem | No.L1900 |
| Alisertib(MLN8237) | SelleckChem | S1133 |
| Puromycin 2HCL | SelleckChem | S7417 |
| Cycloheximide | Sigma-Aldrich | C7698 |
| Melphalan, minimum 95% | Sigma-Aldrich | M2011 |
| Dexamethasone | Sigma-Aldrich | D4902 |
| Carfilzomib | South San Francisco | CA94080 |
| **Enzymes** | | |
| RNase A, DNase and protease-free | Thermo Fisher | EN0531 |
| Proteinase K Solution, ChIP grade | Thermo Fisher | 26160 |
| Benzonase Nuclease | Sigma-Aldrich | E1014-25KU |
| FastAP Thermosensitive Alkaline Phosphatase | Thermo Fisher | EF0651 |
| BsmBI | NewEngland Biolabs | R0580S |
| NotI-HF | NewEngland Biolabs | R3189S |
| BamHI | NewEngland Biolabs | R0136S |
| XbaI | NewEngland Biolabs | R0145S |
| EcoRI | NewEngland Biolabs | R0101S |
| KpnI-HF | NewEngland Biolabs | R3142S |
| HindIII | NewEngland Biolabs | R104S |
| XhoI | NewEngland Biolabs | R0146S |
| CutSmart Buffer | NewEngland Biolabs | 137204S |
| NEBuffer1 | NewEngland Biolabs | B7001S |
| NEBuffer2 | NewEngland Biolabs | B7002S |
| NEBuffer3 | NewEngland Biolabs | B7003O |
| NEBuffer4 | NewEngland Biolabs | B7004S |
| T4 DNA Ligase | NewEngland Biolabs | M0202S |
| 10×Buffer for T4 DNA ligase | NewEngland Biolabs | B0202S |
| Multiscribe Reverse Transcriptase | ABI | 4308228 |
| dNTP mix | ABI | 362275 |
| **Plasmids** | | |
| lentiCRISPRv2 puro | Addgene | |
| pCMV-HA Vector | CLONTECH | |
| lentiCRISPRv2 puro | Addgene | |
| pCMV-HA Vector | CLONTECH | |
| pCMV3-NSD2-HA | Sinobiological | |
| pLV-NSD2-Flag | Sinobiological | |
| pLV-NSD2-Flag (S56D) | Self-construction | |
| pLV-NSD2-Flag (S56A) | Self-construction | |
| PITA-insert | Gift from Dr. Yupeng Chen, Tianjin Medical University | |
| PSPAX_2_ | Gift from Dr. Xudong Wu, Tianjin Medical University, Dept. Cell Biology | |
| PMD_2_G | Gift from Dr. Xudong Wu, Tianjin Medical University, Dept. Cell Biology | |
| pITA-insert-AURKA×FLAG | Self-construction | |
| pITA-insert- AURKA×HA | Self-construction | |
| pITA-insert- AURKA×HA(T288A) | Self-construction | |
| pITA-insert- AURKA×HA(T288D) | Self-construction | |
| AURKA CRISPR1# Custom clone | Self-construction | |
| AURKA CRISPR2# Custom clone | Self-construction | |
| IL6R CRISPR# Custom clone | Self-construction | |
| TCEA2 CRISPR# Custom clone | Self-construction | |
| STC2 CRISPR# Custom clone | Self-construction | |
| NSD2 CRISPR1# Custom clone | Self-construction | |
| NSD2 CRISPR2# Custom clone | Self-construction | |
| **Critical Commercial Assays** | | |
| EvaGreen 2X qPCR MasterMix | ABI | MasterMix-R |
| 5×All-In-One RT MasterMix | abm | G490 |
| Pierce BCA Protein Assay Kit | Thermo SCIENTIFIC | 23225 |
| AxyPrep DNA Extraction Kit | AXYGEN | 295 AP-GX-250G |
| AxyPrep Plasmid Miniprep Kit | AXYGEN | 183 AP-MN-P-250G |
| Plasmid Maxi Kit(25) | QIAGEN | 12163 |
| EnVision G12 Doublestain System,Rabbit/Mouse(DAB+/Permanent Red) | Dako | K5361 |
| SuperSignal West Dura Extended Duration Substrate | ThermoFisher | 34580 |
| Lipofectamine 3000 Transfection Kit | Invitrogen | L3000-008 |
| ANTI-FLAG M2 Affinity Gel | Sigma-Aldrich | A2220 |
| CellTiter 96 Aqueous One Solution | Promega | G358B |
| NuPAGE 4-12% Bis-Tris Gel | Invitrogen | NP0335BOX |
| Phosphatase Inhibitor Cocktail(100×) | Cell Signaling | 587OS |
| Annexin V-FITC Apoptosis Kit | Sigma-Aldrich | APOAF-50TST |
| Pierce® Protein G Plus Agarose | Thermo Scientific | 22852 |
| Ficoll-Paque PLUS endotoxin tested | GE Healthcare | 17-1440-02 |
| LS Columns(25 columns) | Miltenyi Biotec | 130-042-401 |
| TRIzol Reagent | Ambion, Life Science | 15596018 |
| Opti-MEM®I(1×) Reduced Serum | Gibco, Life Technologies | 31985-070 |
| Opti-protein XL Marker | ABM | G266 |
| PageRuler Prestained protein Ladder | ThermoFisher Scientific | 26616 |
| 1Kb Ladder DNA Marker | Biomed | MD114 |
| 1Kb DNA Ladder | TIANCEN | MD111 |
| 100bp DNA Ladder | TRANS | BM301 |
| BM15000 DNA Marker | Biomed | MD106 |
| 1Kb Plus DNA Ladder | Solarbio | M1500 |
| PEI-Transferrinfection Kit | ThermoFisher Scientific | BMS1003 |
| **Primers sequence** | | |
| hGAPDH-F | TTGCCCTCAACGACCACTTT | |
| hGAPDH-R | TGGTCCAGGGGTCTTACTCC | |
| hAURKA-Not1-Flag-F: | ATAAGAATGCGGCCGCatgGATTACAAGGATGACGACGATAAGGACCGATCTAAAG | |
| hAURKA-Kpn1-R: | CGGGGTACCCTAAGACTGTTTGCTAGCTGATT | |
| hAURKA-Not1-HA-F: | ATAAGAATGCGGCCGCatgTACCCATACGATGTTCCAGATTACGCTGACCGATCTAAAG | |
| hAURKA-sgRNA1-Oligo1 | CACCGGCTTGTCTCCAGTCACAAGC | |
| hAURKA-sgRNA1-Oligo2 | AAACGCTTGTGACTGGAGACAAGCC | |
| hAURKA-sgRNA2-Oligo1 | CACCGGTGCTTGCAAAGGAATGCGC | |
| hAURKA-sgRNA2-Oligo2 | AAACGCGCATTCCTTTGCAAGCACC | |
| hAURKA-sgRNA3-Oligo1 | CACCGGTTTACCAGGTGCCGATGGC | |
| hAURKA-sgRNA3-Oligo2 | AAACGCCATCGGCACCTGGTAAACC | |
| hAURKA-sgRNA4-Oligo1 | CACCGGCTAGTTTACCAGGTGCCGATGG | |
| hAURKA-sgRNA4-Oligo2 | AAACCCATCGGCACCTGGTAAACTAGCC | |
| hIL6R-sgRNA-Oligo1 | CACCGTGTGCGTCGCCAGTAGTGTC | |
| hIL6R-sgRNA-Oligo2 | AAACGACACTACTGGCGACGCACAC | |
| hTCEA2-sgRNA-Oligo1 | CACCGAGATTGCGCGGATCGCCCGG | |
| hTCEA2-sgRNA-Oligo2 | AAACCCGGGCGATCCGCGCAATCTC | |
| hSTC2-sgRNA-Oligo1 | CACCGTTGGTCAACGCTGGCGATGT | |
| hSTC2-sgRNA-Oligo2 | AAACACATCGCCAGCGTTGACCAAC | |
| hAIG1-F | GAATCACGGAATGCACACGA | |
| hAIG1-R | GAAAGGGTACACCCACATGC | |
| hAPLP1-F | CCAAGAACCTGCCTAAAGCC | |
| hAPLP1-R | CCAGAGTCTGCAGAATGGAC | |
| hCDKN1A-F | CTTTGTCACCGAGACACCAC | |
| hCDKN1A-R | CAGGTCCACATGGTCTTCCT | |
| hGNG7-F | GGATTGAGCGCATCAAGGTC | |
| hGNG7-R | GGGCATGTTGCTCACAGTAG | |
| hSTC2-F | GAAGCCTGTGCTCCATCTTG | |
| hSTC2-R | CCTGGAGAGCTTGGTTCTGT | |
| hSULF2-F | ATCACGCCGAGCTACAACTA | |
| hSULF2-R | CATGTGGATGGGCTTCATGG | |
| hTCEA2-F | AACTGCACCTACACACAGGT | |
| hTCEA2-R | CAGAACTTCCAGCGGTTTCC | |
| hIL6R-F | TCTCCTGCCAGTTAGCAGTC | |
| hIL6R-R | CAGGCTGCAAGATTCCACAA | |
