# Supplementary material for: Posttranslational modification of Aurora A‐NSD2 loop contributes to drug resistance in t(4;14) multiple myeloma
Source: Clin Transl Med. 2022 Apr 7;12(4):e744. doi: 10.1002/ctm2.744 (PMC8989081; doi:10.1002/ctm2.744)
Supplement: Supplementary file 4 — SUPPORTING INFORMATION [file CTM2-12-e744-s002.docx]

**Table S1** List and characteristics of compounds in the epigenetics compound library
